# Loss of neurofibromin induces inflammatory macrophage phenotypic switch and retinal neovascularization via GLUT1 activation

**DOI:** 10.1016/j.celrep.2025.115625

**Published:** 2025-04-24

**Authors:** Yusra Zaidi, Rebekah Tritz, Nida Zaidi, Faisal Nabi, Syed Adeel H. Zaidi, Abdelhakim Morsy, Valerie Harris, Rilee Racine, Farlyn Z. Hudson, Zsuzsanna Bordan, Simone Kennard, Robert Batori, Yuqing Huo, Gabor Csanyi, Eric J. Belin de Chantemèle, Kecheng Lei, Nicholas M. Boulis, David J. Fulton, Rizwan Hasan Khan, Ruth B. Caldwell, Brian K. Stansfield

**Affiliations:** 1 Vascular Biology Center, Augusta University, Augusta, GA 30912, USA; 2 Department of Pediatrics, Medical College of Georgia, Augusta University, Augusta, GA 30912, USA; 3 James and Jean Culver Vision Discovery Institute, Medical College of Georgia, Augusta University, Augusta, GA 30912, USA; 4 Interdisciplinary Biotechnology Unit, Aligarh Muslim University, Aligarh, UP 202001, India; 5 Department of Ophthalmology, Al-Azhar University, Cairo, Egypt; 6 Department of Cellular Biology and Anatomy, Medical College of Georgia, Augusta University, Augusta, GA 30912, USA; 7 Department of Pharmacology and Toxicology, Medical College of Georgia, Augusta University, Augusta, GA 30912, USA; 8 Department of Medicine, Medical College of Georgia, Augusta University, Augusta, GA 30912, USA; 9 Department of Neurosurgery, Emory University School of Medicine, Atlanta, GA, USA; 10 Department of Biomedical Engineering, Georgia Institute of Technology, Atlanta, GA, USA; 11 Lead contact

## Abstract

Persons with neurofibromatosis type 1 (NF1) exhibit enhanced glucose metabolism, which is replicated in *Nf1-*mutant mice. Inflammatory macrophages invest NF1-associated tumors, and targeting macrophages appears efficacious in NF1 models. Inflammatory macrophages rely on glycolysis to generate ATP; thus, identifying whether neurofibromin, the protein encoded by *NF1*, controls glucose metabolism in macrophages is therapeutically compelling. Using neurofibromin-deficient macrophages and macrophage-specific *Nf1*-knockout mice, we demonstrate that neurofibromin complexes with glucose transporter-1 (GLUT1) to restrain its activity and that loss of neurofibromin permits Akt2 to facilitate GLUT1 translocation to the membrane. In turn, glucose internalization and glycolysis are upregulated and provoke reparative (M^IL4^) macrophages to undergo an inflammatory phenotypic switch. Inflammatory M^LPSIFNγ^ macrophages and inflammatory-like M^IL4^ macrophages invest the perivascular stroma of tumors and induce pathologic angiogenesis in macrophage-specific *Nf1*-knockout mice. These studies identify a mechanism for the enhanced glycolysis associated with NF1 and provide a novel therapeutic target for NF1.

## In brief

Zaidi et al. demonstrate that the loss of neurofibromin in macrophages enhances glucose uptake via membrane-bound GLUT1 in an Akt2-dependent manner, leading to increased glycolysis. This metabolic shift promotes a phenotypic switch from reparative to inflammatory macrophages, resulting in sustained inflammation and pathological neovascularization in retina and human NF1 tumors.

## Graphical Abstract

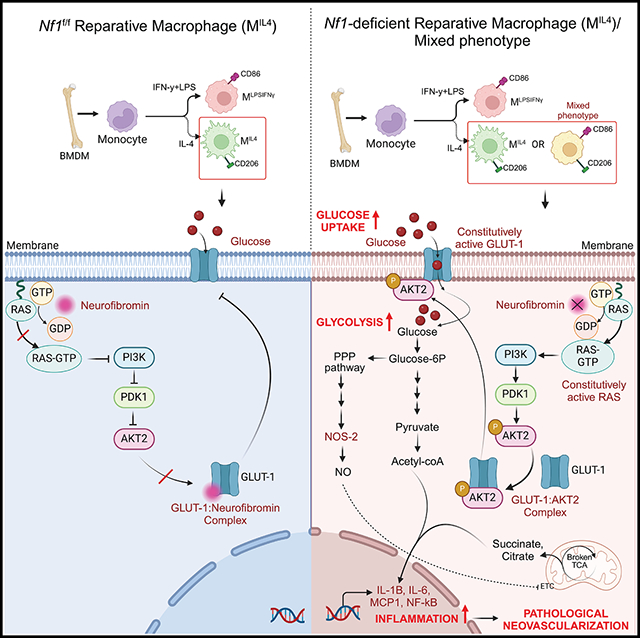

## INTRODUCTION

Neurofibromatosis type 1 (NF1) is a common tumor predisposition syndrome affecting 1 in 2,500 persons worldwide.^1^ Mutations in the *NF1* gene, which causes NF1, have been identified in each of the 60 exons, with more than 2,800 mutations identified to date.^2^ The high mutation rate and size of the *NF1* gene disfavor strong genotype-phenotype correlations. One clear exception to this rule is the highly conserved anthropometric and metabolic phenotype featuring short stature, low body mass index (BMI), and protection from diabetes, which has been described in multiple NF1 cohorts and registry studies.^3–6^ For example, a recently published registry study comparing 1,400 persons with NF1 to 14,000 matched controls revealed a 75% reduction in diabetes prevalence in persons with NF1.^7^ Strikingly, the NF1 cohort was similarly protected from diabetes when compared with non-NF1 siblings, suggesting that *NF1* mutations, but not other genetic or environmental factors, convey this metabolic phenotype. Recently, we demonstrated that mice harboring germline mutations in a single *Nf1* allele (*Nf1*^+/−^) recapitulate many of the metabolic features observed in persons with NF1.^8^ *Nf1*^+/−^ mice rapidly metabolize glucose and are protected from diet-induced hyperglycemia and obesity when compared with littermate wild-type (WT) mice; however, the response to insulin and glucagon did not differ between genotypes, suggesting that neurofibromin, the protein product of *NF1*, controls basal glucose uptake and/or glycolysis. The mechanism through which neurofibromin controls glucose metabolism is unknown.

The increase in basal glucose utilization suggested from observational studies in persons with NF1 and in experimental studies in *Nf1*-mutant mice might be leveraged for novel therapeutics. In this regard, macrophages are particularly attractive targets since functional macrophage populations rely on different metabolic substrates, with “inflammatory” macrophages tending to use glycolysis for the rapid generation of ATP.^9^ This rationale is in keeping with the emergence of clinical and experimental data demonstrating that persons with NF1 and mice harboring *Nf1* mutations experience chronic inflammation characterized by an increased frequency of circulating inflammatory monocytes and cytokines as well as enhanced free radical formation.^10–15^ Macrophages comprise nearly 40% of the total cell mass of NF1 tumors, and targeting macrophage-mediated byproducts has demonstrated efficacy in reducing tumor size, slowing malignant transformation, suppressing arteriopathy, and promoting healthy bone resorption in experimental models of NF1. In many of these models, macrophage subtyping reveals that inflammatory macrophages are the most common population, although other macrophage subpopulations are present, and that they accumulate in the perivascular stroma of tumors and the media of arteries.^15–17^ These observations may be useful for understanding the role of neurofibromin in controlling basal glucose metabolism and macrophage differentiation based on recent data describing enhanced carbohydrate metabolism in persons with NF1 and animals harboring *Nf1* mutations. Whether this highly conserved phenotype may be leveraged for understanding the role of neurofibromin in macrophage polarization and function has not been explored.

Here, we provide genetic and biochemical evidence that neurofibromin interacts with glucose transporter 1 (GLUT1) to maintain GLUT1 in the cytoplasm and that loss of neurofibromin, as seen in persons with NF1, increases membrane-bound GLUT1-mediated glucose uptake. In turn, enhanced glucose uptake in neurofibromin-deficient macrophages induces putative reparative (M^IL4^) macrophages to undergo a phenotypic switch to an inflammatory phenotype and drives pathologic angiogenesis in the retina and human NF1 tumors.

## RESULTS

### Loss of neurofibromin enhances glucose utilization in macrophages

**Figure 1.**
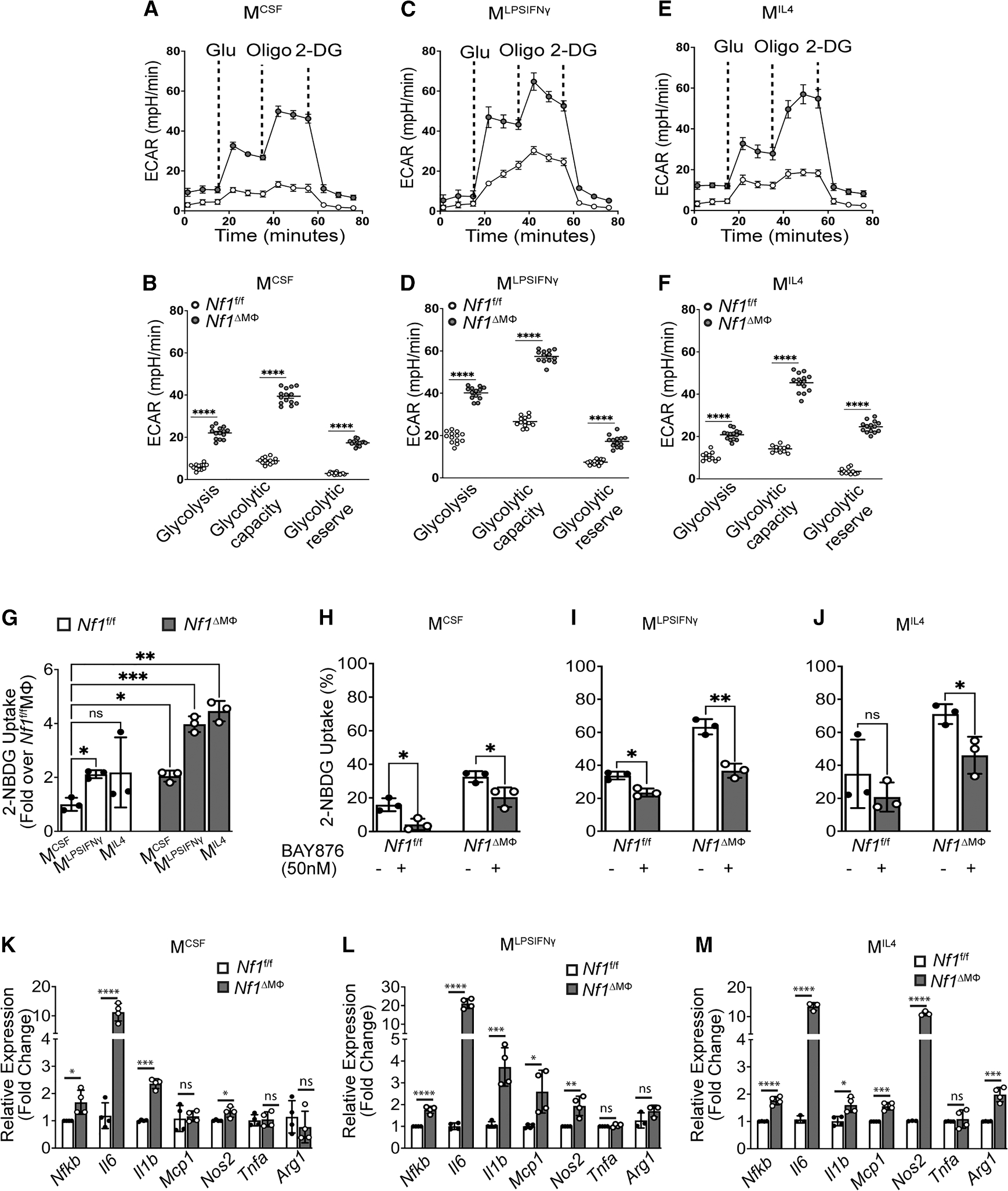
Loss of neurofibromin enhances glycolysis and glucose utilization via GLUT1 in macrophages that results in elevated relative mRNA expression of inflammatory cytokines, NF-κB, and NOS2 and polarizes macrophages to mixed phenotypes (A–F) Representative measurements of extracellular acidification rate (ECAR) performed on bone marrow-derived macrophages (BMDMs) derived from long bones of *Nf1*^f/f^ and *Nf1*^ΔMΦ^ mice treated with 20 ng/mL LPS and IFNγ or 20 ng/mL IL-4 to polarize the macrophages to inflammatory (M^LPSIFNγ^) or reparative (M^IL4^) phenotypes for 16 h, respectively. Subsequent addition of glucose, the ATP synthase inhibitor oligomycin, and the hexokinase inhibitor 2-deoxy-glucose (2-DG) were carried out where indicated (A, C, and E); *n* = 4 mice/genotype; each data point represents technical replicate. Data (mean ± SEM) were normalized by total protein content at the end of experiment. *p* values were calculated using one-way ANOVA followed by Tukey’s multiple comparison t test (*p < 0.05, **p < 0.01, ***p < 0.001, and ****p < 0.0001; ns, not significant). (G–J) Quantification of percentage of glucose uptake by viable BMDMs derived from bones of *Nf1*^f/f^ and *Nf1*^ΔMΦ^ mice in the absence or presence of the GLUT1 inhibitor BAY876. BMDMs were labeled with 2-NBDG at a concentration of 100 μM for 30 min in the absence or presence of BAY876 (50 nM) to measure glucose uptake by flow cytometry. *n* = 3 mice per genotype (also see Figure S3). Data are expressed as mean ± SD. *p* values were calculated using one-way ANOVA followed by Dunnett’s T3 multiple comparison test for (G) and two-tailed Student’s t test for (H)–(J). (K–M) Quantitative RT-PCR analysis of relative mRNA expressions of inflammatory cytokine genes, including *Il6*, *Il1b*, *Mcp1*, *Tnfa*, *Nos2*, and *Nfkb* in (K) control (M^CSF^), (L) inflammatory (M^LPSIFNγ^), and (M) reparative (M^IL4^) macrophages derived from long bones of *Nf1*^f/f^ and *Nf1*^ΔMΦ^ mice. *Rplp0* is considered an endogenous control in this study (*n* = 4 mice per genotype; each data point represents the mean of two technical replicates of each mouse). Data are expressed as mean ± SD. *p* values were calculated using two-tailed Student’s t test (*p < 0.05, **p < 0.01, ***p < 0.001, and ****p < 0.0001; ns, not significant).

Macrophages undergo metabolic reprogramming as they differentiate into inflammatory (M^LPSIFNγ^) and reparative (M^IL4^) phenotypes.^18^ To interrogate the role of neurofibromin in glucose metabolism, we derived macrophages from the long bones of *Nf1*^*flox/flox*^;LysMCre^+^ (*Nf1*^ΔMΦ^) and *Nf1*^*flox/flox*^;LysMCre^−^ (*Nf1*^fl/fl^) mice and exposed them to lipopolysaccharide (LPS) and interferon gamma (IFNγ) to induce an inflammatory (M^LPSIFNγ^) phenotype or interleukin-4 (IL-4) to induce a reparative (M^IL4^) phenotype prior to metabolic characterization. In each macrophage population, *Nf1*^ΔMΦ^ macrophages displayed significantly higher rates of basal glycolysis, glycolytic capacity, and glycolytic reserve when compared with *Nf1*^fl/fl^ macrophages (Figures 1A–1F). A striking feature of these studies was the high glycolytic capacity and reserve of reparative (M^IL4^) *Nf1*^ΔMΦ^ macrophages that approximated the values observed in the traditionally glycolytic inflammatory (M^LPSIFNγ^) population of *Nf1*^ΔMΦ^ macrophages. We observed similar relationships in neurofibromin-deficient human THP1-derived macrophages (Figure S1). We have also observed significant increase in mRNA expression of potent stimulators of glycolysis, hexokinase-1 (*Hk1*) and 6-phosphofructo-2-kinase/fructose-2,6-bisphosphatase (*Pfkfb3*) in both inflammatory (M^LPSIFNγ^) and reparative (M^IL4^) *Nf1*^ΔMΦ^ macrophages as compared to that in *Nf1*^fl/fl^ macrophages (Figure S2). These observations were unique to neurofibromin-deficient MΦs and suggest that loss of neurofibromin enhances glycolysis independent of the polarizing agent.

After observing enhanced glycolysis in each *Nf1*^ΔMΦ^ macrophage population, we sought to identify whether the genotypic differences reflect higher substrate availability. Each *Nf1*^ΔMΦ^ and *Nf1*^fl/fl^ macrophage population was incubated with a glucose analog, 2-deoxy-2-[(7-nitro-2,1,3-benzoxadiazol-4-yl) amino]-D-glucose (2-NBDG) that is readily taken up by cells but fails to undergo the initial steps of glycolysis and accumulates in the cell. In the *Nf1*^fl/fl^ macrophage (i.e., control) populations, glucose uptake mirrored the trends observed in the glycolysis stress test wherein glucose uptake is modestly increased in inflammatory (M^LPSIFNγ^) macrophages and similar glucose internalization was observed between non-polarized and reparative (M^IL4^) macrophages (Figures 1G and S3). As seen in the glycolysis stress test, glucose uptake was significantly increased in all *Nf1*^ΔMΦ^ macrophage populations when compared with each *Nf1*^fl/fl^ macrophage population (Figure 1G). Most striking was the observation that glucose uptake in reparative (M^IL4^) *Nf1*^ΔMΦ^ macrophages approximated and exceeded the glucose uptake observed in inflammatory (M^LPSIFNγ^) *Nf1*^ΔMΦ^ macrophages (Figure 1G). Glucose internalization was inhibited in all *Nf1*^ΔMΦ^ macrophage populations by BAY876, a specific inhibitor of GLUT1, suggesting that basal glucose uptake via GLUT1 accounts for the increase in intracellular glucose and resultant glycolysis observed in *Nf1*^ΔMΦ^ macrophages (Figures 1H–1J and S3).

Since reparative (M^IL4^) macrophages demonstrate similar glucose uptake and glycolysis as inflammatory (M^LPSIFNγ^) macrophages in the absence of neurofibromin, we quantified the mRNA expression of *Nfkb* and a variety of inflammatory cytokines in each macrophage population. Both inflammatory (M^LPSIFNγ^) and reparative (M^IL4^) *Nf1*^ΔMΦ^ macrophages express much higher quantities of mRNA expression of *Il6*, *Il1b*, and *Mcp1* (Figures 1K–1M), which are cytokines that have been linked to NF1 in either human serum or murine models.^13,14,19^ We have also observed increased levels of NOS2 in all *Nf1*^ΔMΦ^ macrophages, but the increase was most dramatic (10-fold) in reparative (M^IL4^) macrophages. Taken together, the results show that the loss of neurofibromin polarizes macrophages toward a pro-inflammatory phenotype irrespective of the polarizing agents.

### Neurofibromin regulates GLUT1 localization to the plasma membrane

**Figure 2.**
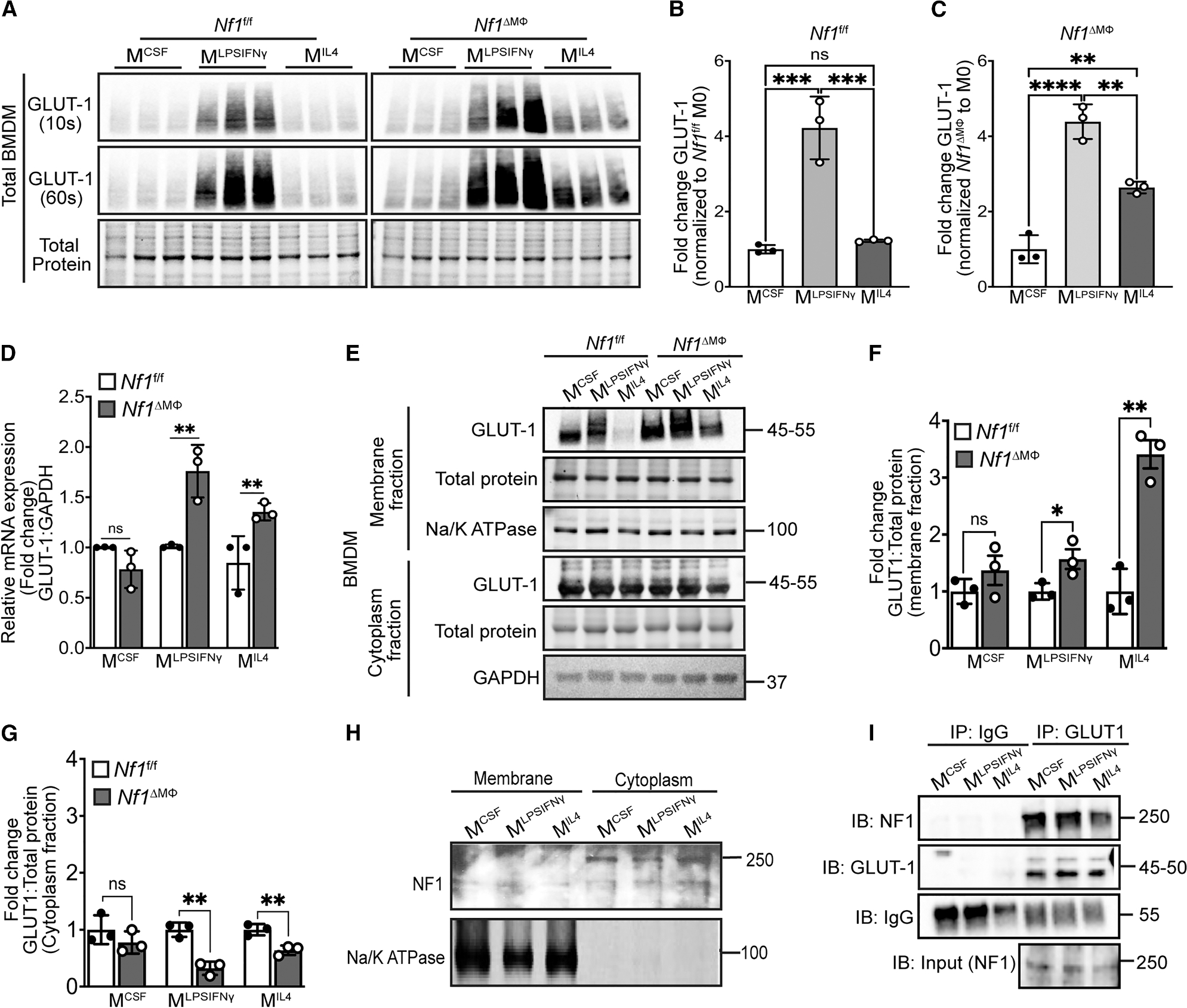
Loss of neurofibromin increases facilitative GLUT1 expression in all macrophages (A–C and E–G) Representative immunoblots and quantification of GLUT1 expression (A–C) in total BMDM lysates and (E–G) in membrane and cytoplasm fractions of BMDMs derived from bones of *Nf1*^f/f^ and *Nf1*^ΔMΦ^ mice. BMDMs were differentiated using macrophage colony-stimulating factor (M-CSF) and polarized to M^LPSIFNγ^ and M^IL4^ macrophages. GLUT1 expression was measured by immunoblotting in all subpopulations of macrophages. *n* = 3 mice per genotype. (D) Quantitative RT-PCR analysis of GLUT1 mRNA relative expression in all subpopulations of macrophages (M^CSF^, M^LPSIFNγ^, and M^IL4^) from bones of *Nf1*^f/f^ and *Nf1*^ΔMΦ^ mice. *n* = 3 mice per genotype; each data point represent the mean of two technical replicates of each mouse. (H) Representatives immunoblot of neurofibromin expression in membrane and cytoplasm fractions of all macrophage subpopulations of *Nf1*^f/f^ mice. (I) Polarized BMDM lysates from *Nf1*^f/f^ mice were prepared and GLUT1 was immunoprecipitated with anti-GLUT1 antibody. The immune complex was then immunoblotted with anti-NF1 antibody to identify the GLUT1:NF1 complex. Data are expressed as mean ± SD. *p* values were calculated using one-way ANOVA followed by Tukey’s multiple comparison test for (B)–(D) and two-tailed Student’s t test for (F) and (G) (*p < 0.05, **p < 0.01, ***p < 0.001, and ****p < 0.0001; ns, not significant).

GLUTs are highly conserved, membrane-bound proteins with varied tissue expression, substrate affinity, and regulatory mechanisms. Within the GLUT family, GLUT1 is ubiquitously expressed, regulates basal glucose uptake independent of insulin, and drives the pro-inflammatory phenotype of macrophages, ensuring sufficient glucose uptake.^20–23^ Based on our previous observations that enhanced glucose utilization in *Nf1*-mutant animals is insulin independent^8^ and present observations that glucose uptake and glycolysis are upregulated in *Nf1*^ΔMΦ^ MΦs, we suspected that GLUT1 expression or function may be altered in *Nf1*^ΔMΦ^ MΦs. As seen in Figure 2A, GLUT1 is predominantly expressed in inflammatory *Nf1*^fl/fl^ macrophages, and a similar but exaggerated increase in GLUT1 was observed in inflammatory (M^LPSIFNγ^) *Nf1*^ΔMΦ^ macrophages. Consistent with the increase in glucose uptake and glycolysis observed in reparative (M^IL4^) *Nf1*^ΔMΦ^ macrophages, we identified a significant increase in GLUT1 expression in reparative (M^IL4^) *Nf1*^ΔMΦ^ macrophages that was not indicative of *Nf1*^fl/fl^ reparative (M^IL4^) macrophages (Figures 2A–2C) with confirmation at the mRNA level (Figure 2D). Next, we isolated the plasma membrane and cytosolic fractions from *Nf1*^ΔMΦ^ and *Nf1*^fl/fl^ macrophages and probed for GLUT1 expression. We observed an increase in GLUT1 in the plasma membrane of inflammatory (M^LPSIFNγ^) *Nf1*^ΔMΦ^ macrophages when compared with inflammatory (M^LPSIFNγ^) *Nf1*^fl/fl^ macrophages (Figure 2E). Consistent with our observations in the whole-cell lysates, membrane-bound GLUT1 was increased over 3-fold in reparative (M^IL4^) *Nf1*^ΔMΦ^ macrophages when compared with reparative (M^IL4^) *Nf1*^fl/fl^ macrophages (Figures 2E and 2F). Further, we observed a reciprocal decrease in cytosolic GLUT1 expression in reparative (M^IL4^) *Nf1*^ΔMΦ^ macrophages that was not observed in reparative (M^IL4^) *Nf1*^fl/fl^ macrophages (Figures 2E and 2G). Taken together, these data suggest that loss of neurofibromin facilitates GLUT1 translocation to the plasma membrane in reparative (M^IL4^) macrophages to mirror the glucose utilization phenotype traditionally observed in inflammatory (M^LPSIFNγ^) macrophages.

Based on the increase in GLUT1 expression in the plasma membrane of *Nf1*^ΔMΦ^ macrophages, we fractionated *Nf1*^fl/fl^ macrophages to identify potential relationships between WT neurofibromin and GLUT1. We identified that neurofibromin is primarily expressed in the cytoplasm of all macrophage populations (Figure 2H). Based on these data and the increased expression of GLUT1 in *Nf1*^ΔMΦ^ macrophages, we hypothesized that neurofibromin interacts with GLUT1 in the cytoplasm of macrophages to restrain GLUT1 from localizing to the plasma membrane in *Nf1*^fl/fl^ macrophages. Therefore, the absence of neurofibromin would allow GLUT1 to localize to the plasma membrane. To interrogate this hypothesis, we precipitated GLUT1 from each *Nf1*^fl/fl^ macrophage population and probed for neurofibromin. As seen in Figure 2I, neurofibromin co-precipitates with GLUT1 in all *Nf1*^fl/fl^ macrophage populations. We conclude that loss of neurofibromin leads to unrestrained GLUT1 translocation to the plasma membrane of macrophages and permits glucose internalization and utilization.

### Loss of neurofibromin increases GLUT1 expression via Akt2

**Figure 3.**
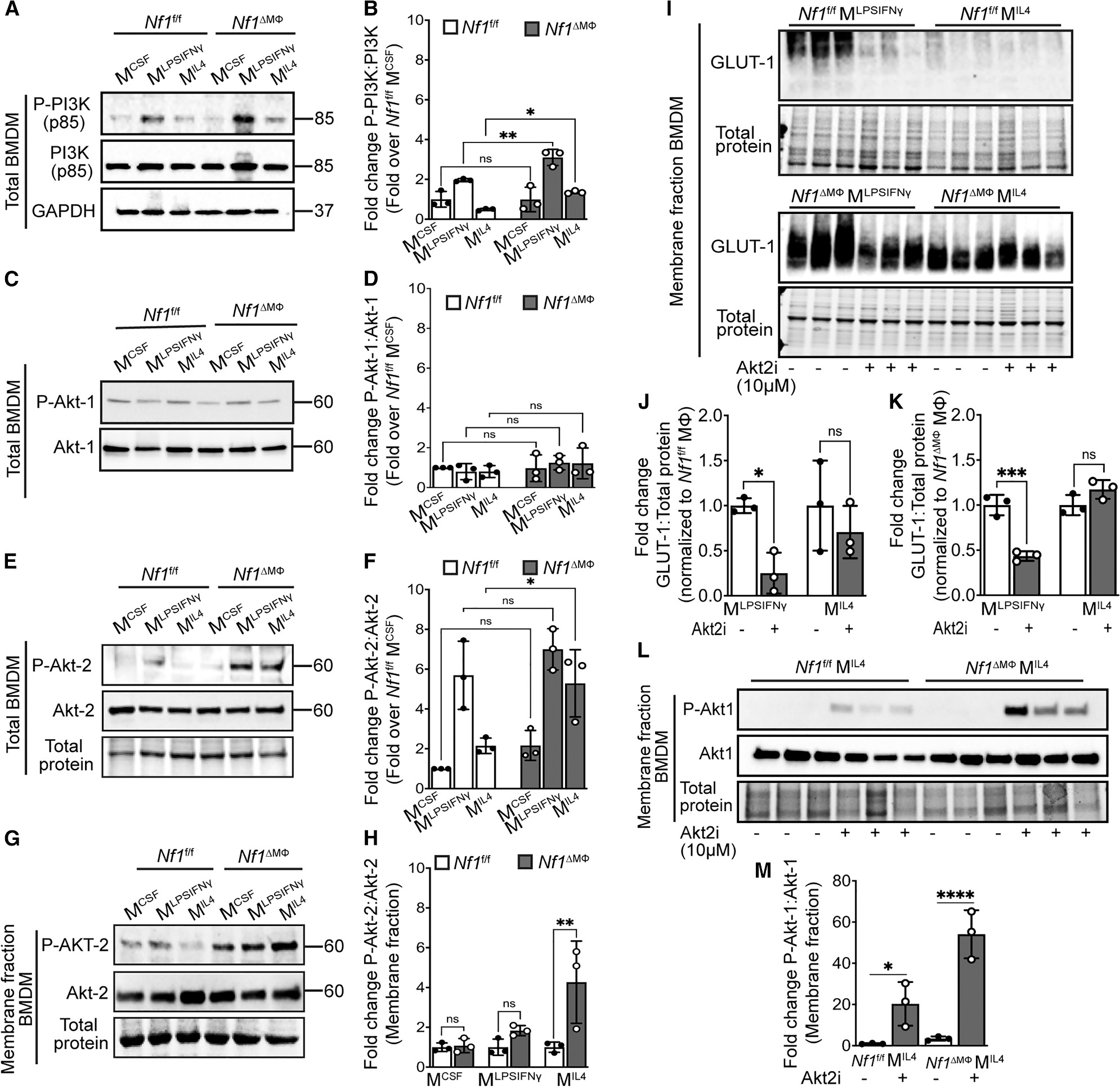
Loss of neurofibromin preferentially increases Akt-2 phosphorylation, but not Akt1 phosphorylation, in total and in membrane fractions of all macrophage subpopulations and promotes GLUT1 translocation to the cell membrane via Akt2 (A–H) Representative immunoblots and densitometry graphs showing the ratios of (A and B) P-PI3K/PI3K, (C and D) P-Akt-1/Akt-1, (E and F) P-Akt-2/Akt-2 expression in total BMDM lysates, and (G and H) P-Akt-2/Akt-2 expression in membrane fractions of BMDMs from long bones of *Nf1*^f/f^ and *Nf1*^ΔMΦ^ mice. *n* = 3 mice per genotype. Data are expressed as mean ± SD. *p* values were calculated using one-way ANOVA followed by Sidak’s multiple comparison test for (B), (D), (F), and (H) (*p < 0.05, **p < 0.01, ***p < 0.001, and ****p < 0.0001; ns, not significant). (I–M) Representative immunoblots and densitometry graphs showing (I–K) GLUT1 expression in membrane fractions of both inflammatory (M^LPSIFNγ^) and reparative macrophages (M^IL4^) and (L and M) P-Akt-1 and total Akt-1 expression in membrane fractions of only M^IL4^ macrophages from long bones of *Nf1*^f/f^ and *Nf1*^ΔMΦ^ mice after Akt2 inhibitor (Akt2i; CCT128930) treatment at a dose of 10 μM for 6 h (*n* = 3 per genotype). Data are expressed as mean ± SD. *p* values were calculated using one-way ANOVA followed by Sidak’s multiple comparison test (*p < 0.05, **p < 0.01, ***p < 0.001, and ****p < 0.0001; ns, not significant).

The increase in membrane-bound GLUT1 in the absence of neurofibromin explains the enhanced glucose uptake and glycolysis observed in *Nf1*^ΔMΦ^ macrophages, but we also observed an increase in the expression of GLUT1 in whole *Nf1*^ΔMΦ^ macrophage lysates. Neurofibromin is principally recognized as a regulator of the Ras-Mek-Erk pathway, which is the focus of several inhibitor agents for persons with NF1, but Ras also regulates pathways linked to GLUT expression, such as PI3-kinase (PI3K) and Akt. Therefore, we probed whole-cell lysates from each *Nf1*^ΔMΦ^ and *Nf1*^fl/fl^ macrophage population and identified the activation of PI3K (phosphorylated PI3K [P-PI3K]) and both Akt isoforms in inflammatory (M^LPSIFNγ^) and reparative (M^IL4^) *Nf1*^ΔMΦ^ macrophages (Figures 3A–3F). Consistent with the increase in GLUT1 expression in reparative (M^IL4^) *Nf1*^ΔMΦ^ macrophages, the expression of P-PI3K and P-Akt2, but not P-Akt1, is significantly higher in reparative (M^IL4^) *Nf1*^ΔMΦ^ macrophages when compared to reparative (M^IL4^) *Nf1*^fl/fl^ macrophages (Figures 3A–3F). To confirm this relationship, we probed the membrane fraction of all macrophage populations from both genotypes for Akt2 and identified a 4-fold increase in P-Akt2 in reparative (M^IL4^) *Nf1*^ΔMΦ^ macrophages (Figures 3G and 3H). These data suggest that loss of neurofibromin induces GLUT1 expression through Akt2 activation.

To determine the relationship between Akt2 and GLUT1 in the absence of neurofibromin, we analyzed the membrane fraction of inflammatory (M^LPSIFNγ^) and reparative (M^IL4^) *Nf1*^ΔMΦ^ and *Nf1*^fl/fl^ macrophages in the presence of CCT128930, a selective Akt2 inhibitor. Pharmacologic inhibition of Akt2 completely suppressed GLUT1 translocation to the plasma membrane of inflammatory (M^LPSIFNγ^) *Nf1*^fl/fl^ macrophages, with similar but diminished results seen in inflammatory (M^LPSIFNγ^) *Nf1*^ΔMΦ^ macrophages (Figures 3I–3K). In comparison, GLUT1 expression in the plasma membrane of reparative (M^IL4^) *Nf1*^ΔMΦ^ macrophages was not altered in the presence of an Akt2 inhibitor. This surprising observation led us to probe the membrane lysates of reparative (M^IL4^) macrophages from both genotypes for P-Akt1 to identify potential compensatory mechanisms resulting from pharmacologic inhibition of Akt2. In fact, we identified that Akt2 inhibition increased the expression of P-Akt1 in both genotypes of reparative (M^IL4^) macrophages, but the increase was much more substantial in the reparative (M^IL4^) *Nf1*^ΔMΦ^ macrophages (Figures 3L and 3M). Given the relatively low expression of GLUT1 in reparative (M^IL4^) *Nf1*^fl/fl^ macrophages, we conclude that the impact of Akt2 inhibition on Akt1 activation has little impact on GLUT1 localization in the presence of neurofibromin. Conversely, the loss of neurofibromin accentuates Akt1 signaling as a compensatory pathway when Akt2 is inhibited to maintain the high expression of active GLUT1 in the plasma membrane.

### Neurofibromin complexes with GLUT1 and conserved regions of Akt2

**Figure 4.**
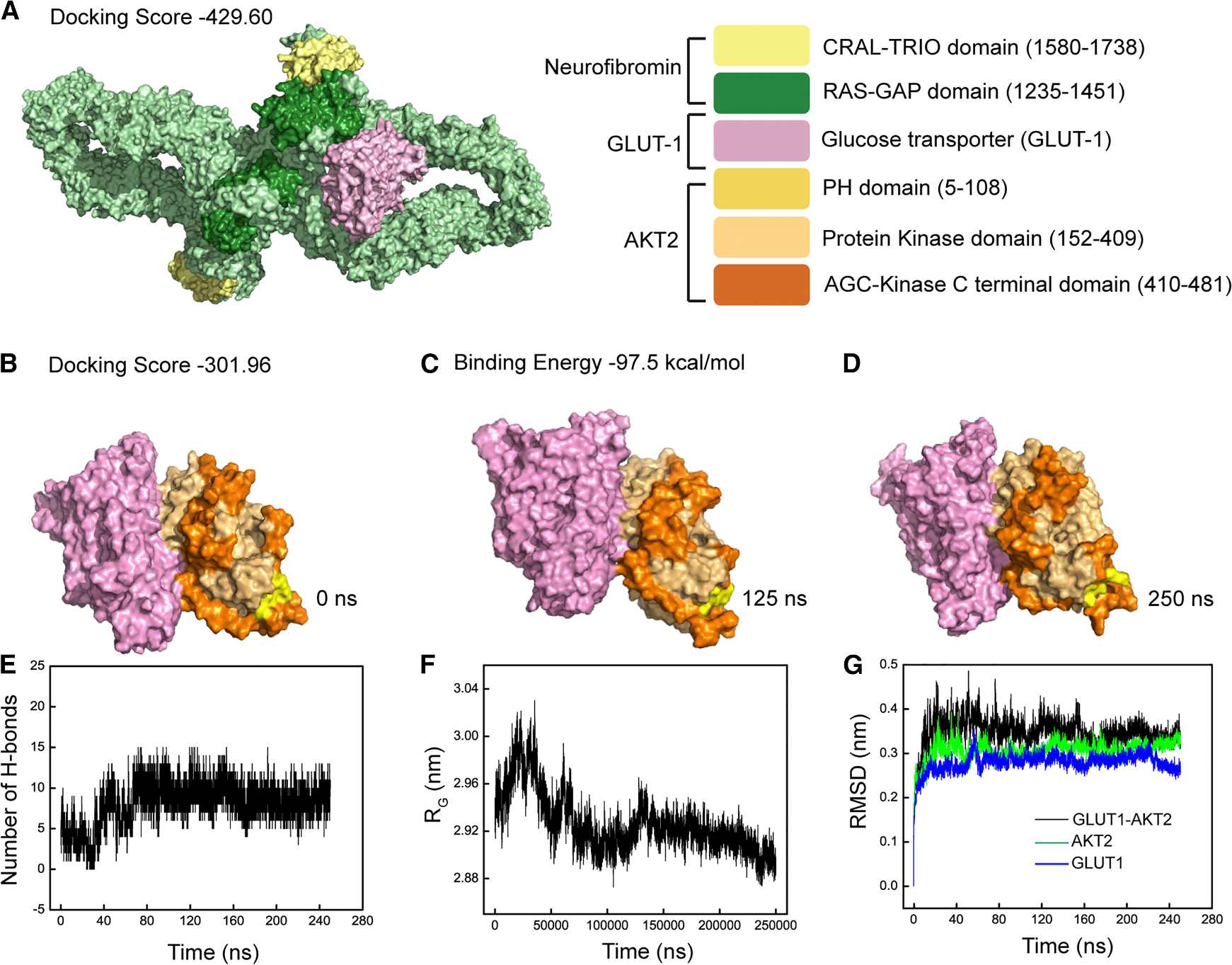
Neurofibromin complexes with GLUT1 and conserved regions of Akt2 Molecular docking and atomistic simulations implicate the complex formation of NF1-GLUT1 and GLUTI-Akt2. (A–D) Surface representations of (A) NF1-GLUT1 complex having docking score of −429.60 obtained by H-dock, (B) GLUT1-Akt2 at 0 ns with docking score of −301.96, (C) GLUT1-Akt2 at 125 ns, and (D) GLUT1-Akt2 at 250 ns with binding energy of −97.5 kcal/mol obtained by simulations. (E–G) Plots showing (E) number of H-bonds present in GLUT1-Akt2 complex over a period of 250 ns, (F) radius of gyration (RG) of GLUT1-Akt2 complex over a period of 250 ns, and (G) root-mean-square deviation (RMSD) of GLUT1, Akt2, and GLUT1-Akt2 complex (also see Table S1).

Given the compensatory effects of Akt1 in the presence of an Akt2 inhibitor, we examined the interaction between GLUT1 and neurofibromin or Akt2 by using the crystal structures of neurofibromin, GLUT1, and Akt2 to perform protein-protein docking, molecular dynamics simulation, and identify the docking score and binding affinity between molecules. Figure 4A shows the cartoon representation of neurofibromin docked with GLUT1 and Figure 4B shows GLUT1 docked with Akt2 with docking scores of −429.60 and −301.96, respectively. The binding of GLUT1 with neurofibromin is likely to be stronger than its binding with Akt2 based on their respective docking scores, indicating a more plausible binding model for neurofibromin and GLUT1 when compared with GLUT1 and Akt2. Although GLUT1 docking with neurofibromin is adjacent to the Ras-GAP domain (residues 1235–1451) in this model, few amino acid residues in this domain actively interact with residues of GLUT1 (Table S1). GLUT1 binds to Akt2 via the protein kinase and AGC-kinase C-terminal domain that may be independent of its binding to neurofibromin since only four amino residues, i.e., ARG89, PHE90, MET98, and LEU101 (highlighted in Table S1), of GLUT1 are predicted to be shared between the two complexes. Two residues in GLUT1 (ARG89 and PHE90) are predicted to actively interact with both Akt2 domains. Given the more plausible binding model for neurofibromin and GLUT1, Akt2 has a lower propensity to complex with GLUT1 in the presence of neurofibromin. Conversely, the absence of neurofibromin would increase the propensity for GLUT1 to interact with Akt2 based on the docking model.

To determine the stability of the GLUT1-Akt2 complex, atomistic simulations were generated for 250 ns, with the conformations of the complex at 0, 125, and 250 ns shown in Figures 4B–4D, respectively. The simulations estimate that the GLUT1-Akt2 complex is strong with a binding energy of −97.50 kcal/mol, which strengthened over time with an increase in H-bonds from 5 to 11 with consistent radii of gyration values lying between 2.89 and 2.92 nm (Figures 4E and 4F). Finally, we assessed the conformational drift of Akt2, GLUT1, and the GLUT1-Akt2 complex by calculating the root-mean-square deviation (RMSD) of atomic positions. Equilibration of the GLUT1Akt2 complex was reached at ~160 ns in simulations and did not fluctuate (Figure 4G). Further, the overall RMSD or equilibrium value for the complex is higher than either Akt2 or GLUT1, suggesting a predictable strong and stable complex in the absence of neurofibromin.

### Loss of neurofibromin in macrophages is sufficient to induce retinal neovascularization

**Figure 5.**
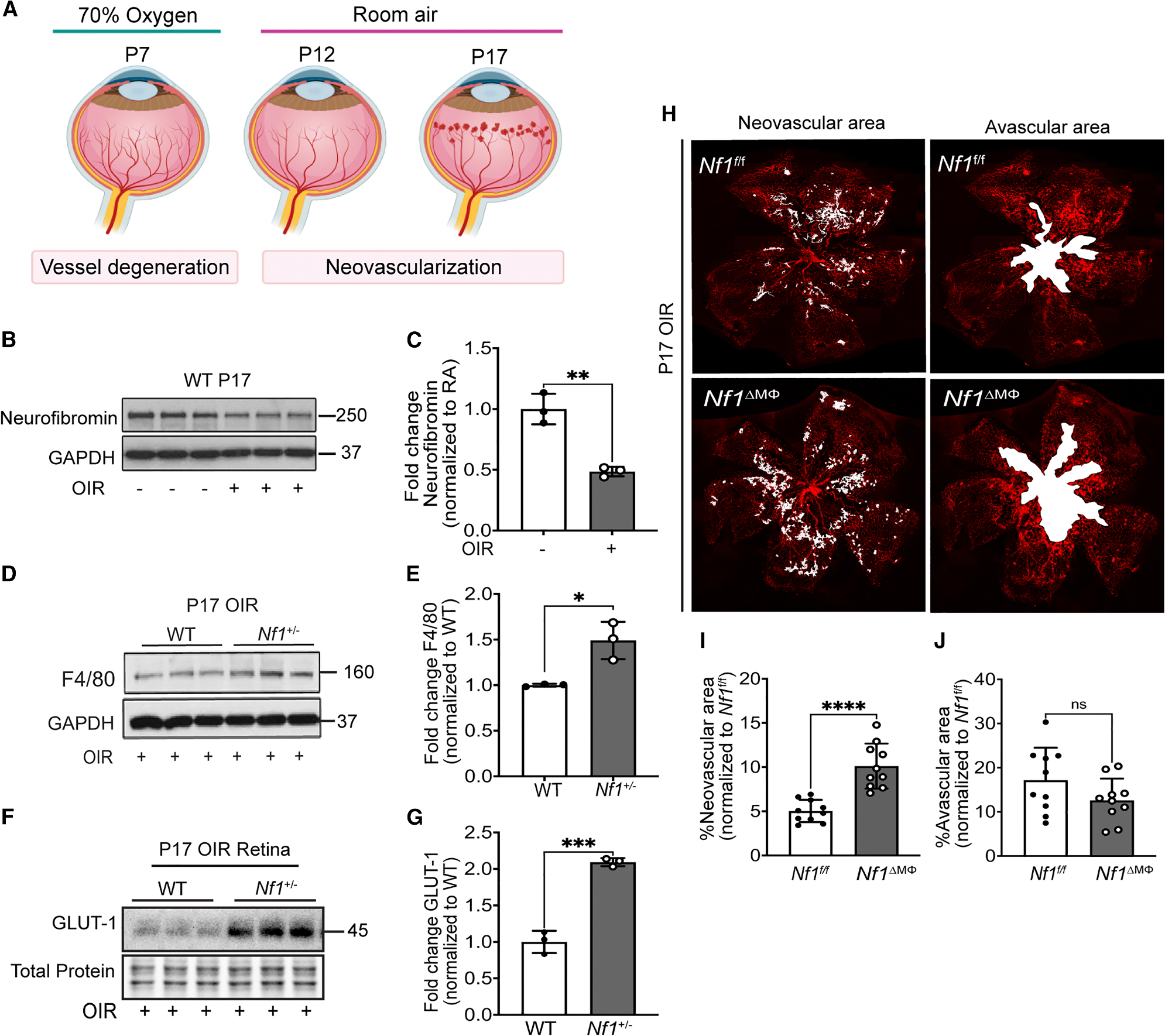
Neurofibromin is sensitive to hypoxia, and loss of neurofibromin in macrophages/microglia is sufficient to induce pathological retinal neovascularization (A) Schematic of the mouse oxygen-induced retinopathy (OIR) model. (B–G) Representative immunoblots and quantifications showing (B and C) neurofibromin expression in P17 retinas from WT mice exposed to room air (−) or OIR (+) (*n* = 3 mice per group), (D and E) F4/80 expression in P17 retinas from WT and *Nf1*^+/−^ mice exposed to OIR (*n* = 3 mice per genotype), and (F and G) upregulation of GLUT1 expression in P17 retinas from WT and *Nf1*^+/−^ mice exposed to OIR (*n* = 3 mice per genotype). (H) Representative retinal whole mounts from P17 OIR retinas of *Nf1*^f/f^ and *Nf1*^ΔMΦ^ mice stained with isolectin-B4 (red) with highlighted neovascular areas and avascular areas (white). (I and J) Quantification of neovascular areas and avascular areas in OIR retinas were expressed as percentage of total retinal areas (*n* = 10 retinas). Data are expressed as mean ± SD. *p* values were calculated using two-tailed Student’s t-test (*p < 0.05, **p < 0.01, ***p < 0.001, and ****p < 0.0001; ns, not significant).

Characteristic retinal neovascular lesions are observed in ~80% of persons with NF1 and are reproduced in mice harboring germline *Nf1* mutations.^24^ Given the metabolic phenotype observed in persons with NF1 and evidence of chronic inflammation in this population, we sought to identify whether loss of neurofibromin in macrophages and subsequent inflammatory phenotypic switch might induce retinal neovascularization. Initially, we subjected WT and *Nf1* heterozygous (*Nf1*^+/−^) mice to oxygen-induced retinopathy (OIR; Figure 5A) to demonstrate that neurofibromin expression is reduced in WT retina lysates and that F4/80 expression, a putative macrophage marker, is increased in *Nf1*^+/−^ retinas subjected to OIR (Figures 5B–5E). We also demonstrate that *Nf1*^+/−^ retinas exhibit substantially more GLUT1 when compared with littermate WT retinas subjected to OIR (Figures 5F and 5G). Next, we subjected *Nf1*^ΔMΦ^ and *Nf1*^fl/fl^ mice to OIR and identified a significant increase in neovascular area in *Nf1*^ΔMΦ^ retinas when compared with *Nf1*^fl/fl^ retinas subjected to OIR, suggesting that the loss of neurofibromin in macrophages is sufficient to induce retinal neovascularization and recapitulates a feature of human NF1 (Figures 5H and 5J).

**Figure 6.**
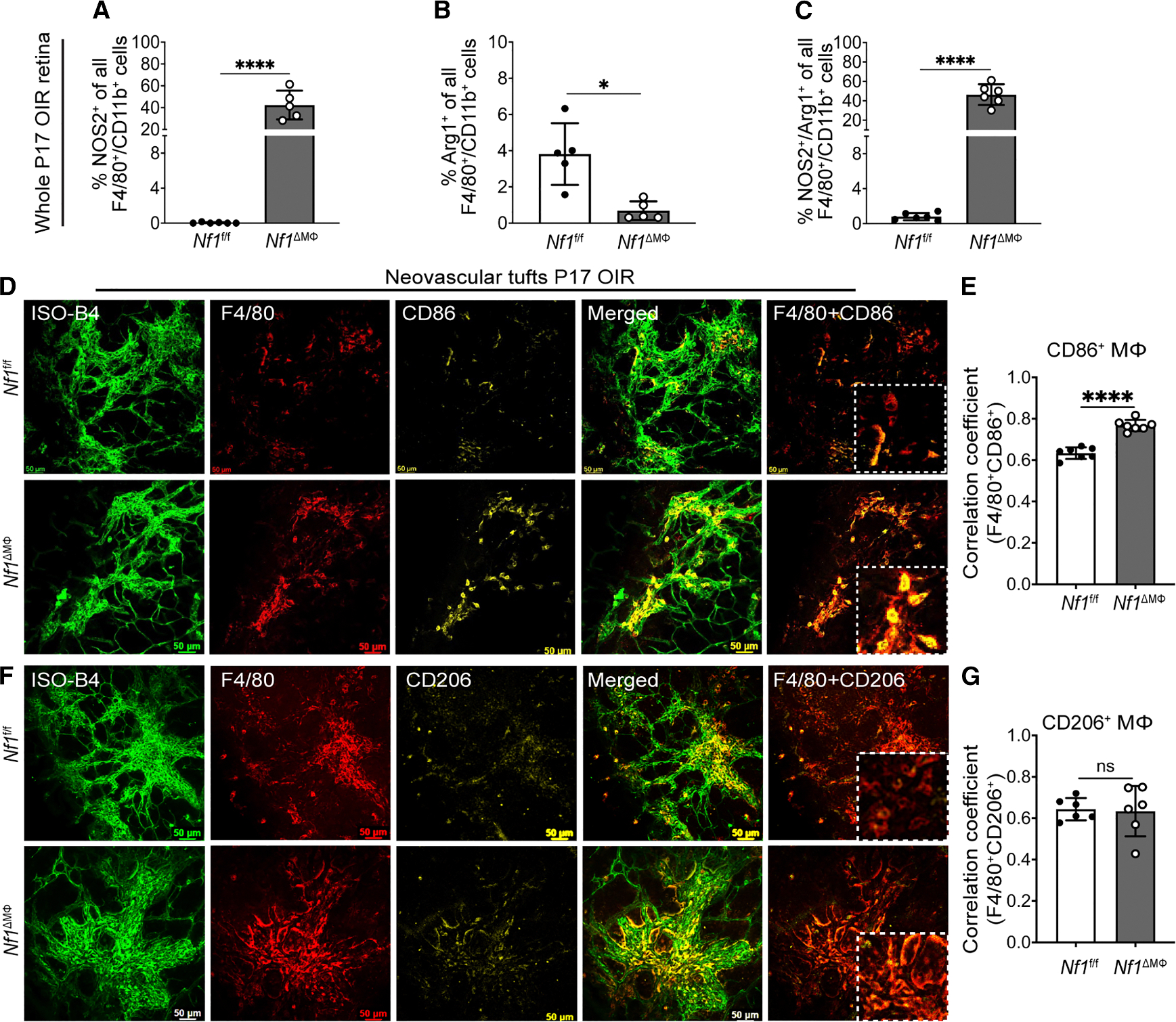
Loss of neurofibromin promotes inflammatory macrophage/microglia infiltration and localization to sites of neovascular tufts in the retina (A–C) Flow cytometric analysis of whole P17 retinas from *Nf1*^f/f^ and *Nf1*^ΔMΦ^ mice exposed to OIR-expressing macrophage or microglial markers (also see Figure S4). Plots represent the percentages of viable (A) inflammatory macrophage/microglia (F4/80^+^/CD11b^+^/NOS2^+^), (B) reparative macrophage/microglia (F4/80^+^/CD11b^+^/Arg1^+^), and (C) macrophage/microglia with mixed phenotype (F4/80^+^/CD11b^+^/NOS2^+^/Arg1^+^) in P17 OIR retinas (*n* = 5–6 mice per genotype; total of 10–12 retinas in each genotype). (D and F) Confocal images of retinal flat mounts at P17 OIR from *Nf1*^f/f^ and *Nf1*^ΔMΦ^ mice, stained with isolectin-B4 (vessel stain), F4/80, and either (D) CD86 (inflammatory macrophage/microglia) or (F) CD206 (reparative macrophage/microglia) to show the localization of macrophage/microglia near neovascular tufts. (E and G) Pearson’s correlation coefficient for F4/80 with either (E) CD86 or (G) CD206 to depict co-localization (*n* = 6 retinas in each group). Scale bars: 50 μm (D and F) and 10 μm (inset). Data are expressed as mean ± SD. *p* values were calculated using two-tailed Student’s t test (*p < 0.05, **p < 0.01, ***p < 0.001, and ****p < 0.0001; ns, not significant).

Next, we used flow cytometry and immunohistochemistry to identify inflammatory and reparative macrophages within *Nf1*^ΔMΦ^ and *Nf1*^fl/fl^ postnatal day (P)17 retinas subjected to OIR using the putative markers NOS2 (i.e., inducible nitric oxide synthase [iNOS]) to identify inflammatory (M^LPSIFNγ^) macrophages and arginase-1 to identify reparative (M^IL4^) macrophages. We identified that NOS2-expressing retinal macrophages/microglia were increased 40-fold in P17 OIR *Nf1*^ΔMΦ^ retinas when compared with *Nf1*^fl/fl^ retinas (Figure 6A). Retinal macrophages/microglia expressing arginase-1 were significantly reduced in P17 OIR *Nf1*^ΔMΦ^ retinas when compared with P17 OIR *Nf1*^fl/fl^ retinas (Figure 6B). Although arginase-1-expressing macrophage/microglia were rarely identified in the retinas from either genotype subjected to OIR, the number of CD11b^+^/F4/80^+^ retinal macrophages/microglia that co-express NOS2 and arginase-1 was increased ~40-fold in P17 OIR *Nf1*^ΔMΦ^ retinas when compared with P17 OIR *Nf1*^fl/fl^ retinas (Figures 6C and S4). These results mirror the large increase in NOS2 and arginase-1 identified in reparative (M^IL4^) neurofibromin-deficient macrophages in Figure 1M. Finally, we examined neovascular tufts from *Nf1*^ΔMΦ^ and *Nf1*^fl/fl^ P17 retinas subjected to OIR to identify inflammatory and reparative perivascular macrophages. Similarly, we identified that inflammatory CD86-expressing macrophages were more common in the neovascular tufts of *Nf1*^ΔMΦ^ retinas when compared with *Nf1*^fl/fl^ retinas subjected to OIR, but no difference in reparative CD206 macrophage content was identified in P17 OIR retinas from both genotypes (Figures 6D–6G). Taken together, the results show that the loss of neurofibromin in macrophages facilitates the accumulation of perivascular inflammatory (M^LPSIFNγ^) macrophages to promote retinal neovascularization. A significant proportion of these retinal inflammatory (M^LPSIFNγ^) macrophages co-express putative reparative markers such as arginase-1, suggesting that loss of neurofibromin induces an inflammatory phenotypic switch of reparative (M^IL4^) macrophages.

### GLUT1 and phosphorylated-Akt2 co-localize within macrophages from NF1-associated tumors

**Figure 7.**
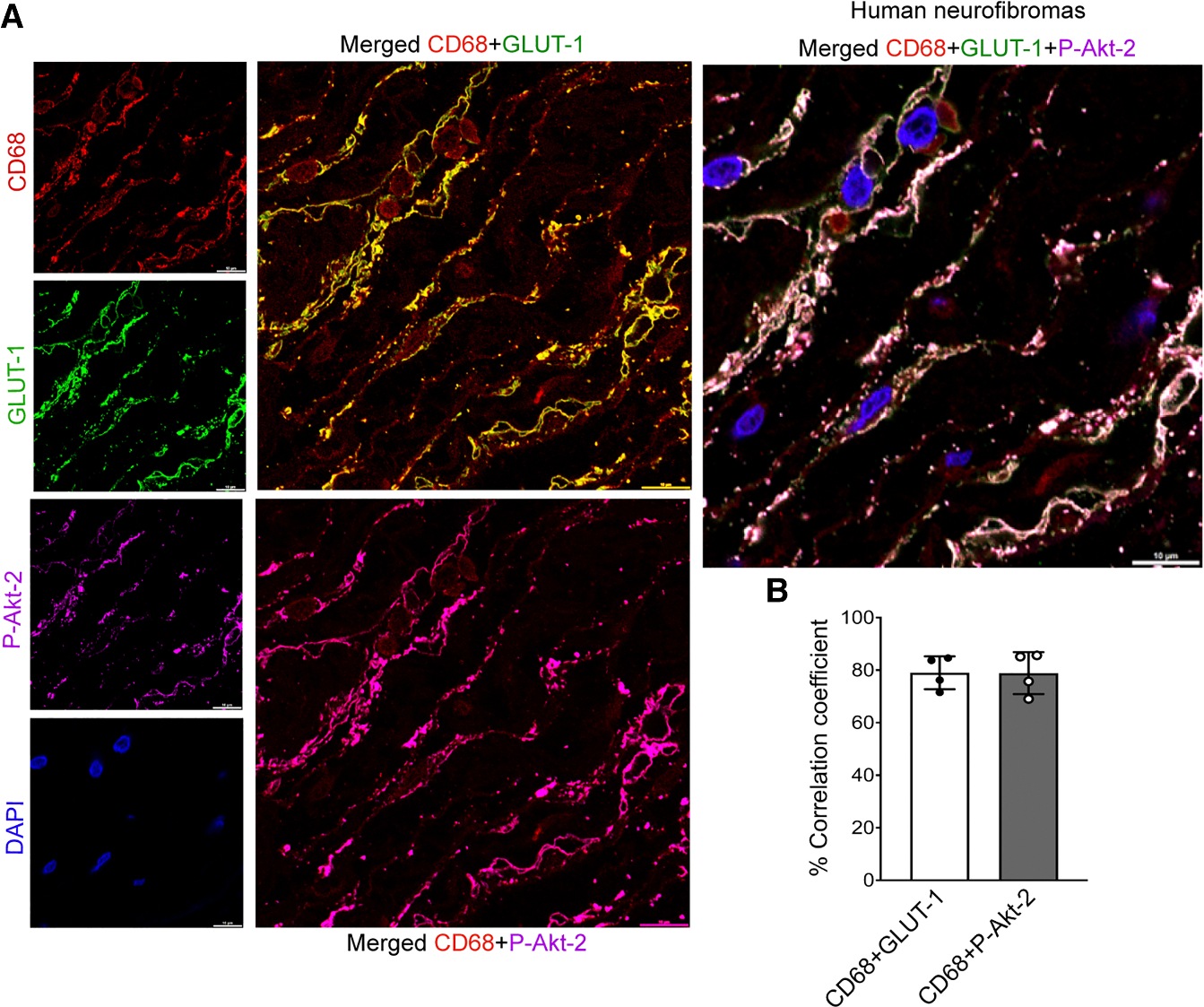
Loss of neurofibromin results in GLUT1 and Akt2 co-localization within CD68^+^ macrophages from NF1-associated tumors (A) Representative high-magnification confocal images of NF1-associated tumor cross-section from human patients showing co-localization of GLUT1 and P-Akt2 with CD68^+^ macrophages, Scale bars: 10 μm. (B) Percentage of Pearson’s correlation coefficient for CD68 with either GLUT1 or P-Akt2 to depict co-localization (*n* = 4 patients with NF1). Each data point reflects the values to each patient (also see Table S2).

It is well documented that macrophages are prevalent in neurofibromas and malignant peripheral nerve sheath tumors (MPNSTs) and correlate with disease progression.^25–27^ However, the reason why macrophage density increases with disease progression is still not well explained. Therefore, we sought to confirm our observations from lineage-restricted *Nf1*-mutant mice in relevant human tissue. Procuring human retina tissue from persons with NF1 is not practical; therefore, we examined tumor samples from persons with NF1 for expression of GLUT1 and P-Akt2 within perivascular macrophages. We identified 5 plexiform neurofibroma (pNF) and MPNST samples from 4 patients (mean age = 31 years, 50% male; details in Table S2) and probed paraffin-embedded sections for GLUT1 and P-Akt2. Both GLUT1 and P-Akt2 co-localized with 80% of the CD68-expressing macrophages within these tumor samples, suggesting a high degree of GLUT1 activation in pNF- and MPNST-associated macrophages (Figures 7A and 7B).

## DISCUSSION

NF1 is a multi-system disease resulting from dominantly inherited mutations in the *NF1* tumor-suppressor gene, which encodes neurofibromin. Despite the relative inconsistency in the timing and penetrance of any one manifestation in persons with NF1, human cohort studies have identified a characteristic metabolic and anthropometric phenotype characterized by short stature and reduced adiposity, as well as strong protection from insulin resistance and diabetes. A recent registry study of nearly 2,500 persons with NF1 demonstrated that persons with NF1 have statistically lower odds of hospitalization for endocrine-related diseases when compared with the general population.^28^ Of the 11 main diagnostic categories for hospitalization days, only endocrine-related diagnoses produced a negative association with NF1. Interestingly, the risk for endocrine-related hospitalization was reduced by 80% and 40% in males and females with NF1, respectively. When examining individual diagnoses within this category, the prevalence of diabetes mellitus type 1 was reduced 2.5-fold, while diabetes mellitus type 2 prevalence was similar to the general population. A more recent Finnish registry study explored this potential relationship with a specific focus on diabetes mellitus.^7^ In comparison to non-NF1 siblings and non-related matched controls, the hazard ratios for diabetes mellitus type 1 in the NF1 cohort were 0.55 and 0.58, respectively. Again, males with NF1 seem to display greater protection from diabetes mellitus type 1 when compared with male siblings and male controls with hazard ratios of 0.26 and 0.29, respectively. Finally, those persons with NF1 who were diagnosed with diabetes mellitus type 1 appear to require less insulin when compared with sibling and non-sibling controls. Experimental human studies identified a nearly 90% lower risk of persons with NF1 having a fasting blood glucose level above 100 mg/dL when compared with matched non-NF1 controls but no group differences in insulin or blood glucose values after administration of a dextrose bolus.^5,6^ We have demonstrated similar findings in *Nf1* heterozygous mice.^8^ Taken together, these human registry data and experimental studies are highly suggestive that mutations in the *NF1* gene harbor a hormone-independent relationship to enhanced glucose internalization and/or metabolism, which serves as the theoretical basis for our experiments.

Our long-standing interest is understanding why persons with NF1 experience chronic inflammation and the untoward effects of acute and sustained inflammation on this population. Macrophages are a rational population for mechanistic studies in NF1 and are pathogenically implicated in multiple models of NF1. Human and murine NF1/Nf1 tumors are replete with macrophages, and macrophage density correlates with tumor growth and malignant transformation.^15,25,27,29^ Descriptive studies have consistently pointed to an inflammatory signature in these tumors with a preponderance of so-called inflammatory macrophages and high expression of inflammatory cytokines corresponding to a lower-than-expected number of so-called reparative macrophages. The 10- to 20-fold increase in secreted IL-6 from neurofibromin-deficient macrophages is of particular interest, as several publications have demonstrated higher plasma IL-6 concentrations in persons with NF1 when compared with matched controls.^13,14,30^

Here, we converge these observations to demonstrate that loss of neurofibromin does impart a rapid and sustained entrainment of glucose into macrophages that is mediated through GLUT1. We also demonstrate that the relationship between GLUT1 and neurofibromin is complex within these cells such that the increase in membrane-bound GLUT1 in neurofibromin-deficient cells is not limited to so-called inflammatory (M^LPSIFNγ^) macrophages, which are highly reliant on GLUT1 to internalize glucose. Rather, we identified that so-called reparative (M^IL4^) macrophages also exhibit high membrane-bound GLUT1 in the absence of neurofibromin, corresponding to a reduction in cytoplasm GLUT1 content. This imbalance in functional GLUT1 availability may skew the so-called reparative macrophages to exhibit features that are more commonly identified in inflammatory macrophages. In keeping with this line of thinking, both inflammatory and reparative neurofibromin-deficient macrophages express much higher inflammatory cytokine content than WT macrophages. Further, we demonstrate that loss of neurofibromin in macrophages alone is sufficient to induce retinal neovascularization and that the neovascular tufts are invested with inflammatory macrophages. Again, we find that retinal macrophages in *Nf1*^ΔMΦ^ retinas subjected to OIR are not dichotomous, with some macrophages expressing putative markers of both macrophage subpopulations. While this same phenomenon is seen in control retinas, it is much less pronounced and further supports our hypothesis that loss of neurofibromin promotes both classic inflammatory macrophage function and phenotypic switching of classic reparative macrophages.

The ability of neurofibromin-deficient macrophages to take on properties of both inflammatory and reparative macrophages provides insight into many manifestations of NF1 that have been linked to inflammatory changes, cells, or cytokines in animal models or human tissue. It has been speculated that macrophages are part of a secondary response to a disease-initiating event and propagate pathology. For example, multiple investigative teams have demonstrated that inflammatory macrophages are abundant in NF1 tumors and that primary tumor cells secrete growth factors and cytokines that recruit both WT and neurofibromin-deficient macrophages toward the stimulus.^16,27,31^ In line with this paradigm, we have demonstrated that the increased sensitivity to growth factors and cytokines observed in neurofibromin-deficient macrophages may extend beyond a mere signaling amplification to include an increased propensity to sample their extracellular environment through endocytosis.^10,32,33^ However, this line of thinking does not elucidate the fundamental question of why inflammatory macrophages are abundant in tumors and other manifestations of NF1. Here, we identify a conserved feature of neurofibromin-deficient macrophages and tissue that enhanced glucose internalization, via a constitutively active GLUT1, underlies these previously recognized features. Given this defining feature of inflammatory macrophages and experimental evidence suggesting their role in driving NF1-related pathologies such as tumor growth and malignant transformation, targeting glucose internalization or glycolysis specifically in macrophages is an attractive therapeutic approach for persons with NF1.

Targeting GLUT1 directly is not a simple prospect owing to its necessity for maintaining the basal glucose supply to most cells. We demonstrate that membrane-bound GLUT1 is controlled by Akt2, but loss of neurofibromin complicates this relationship, particularly in so-called reparative (M^IL4^) macrophages. In the presence of neurofibromin, Akt2 binding to GLUT1 is impaired, which likely explains the clear loss of membrane-bound GLUT1 following Akt2 inhibition. When neurofibromin is absent, GLUT1 is more likely to bind to Akt2 based on inhibitor studies and computational-based models, which, in turn, enhances GLUT1 expression in the membrane and explains the increase in glucose internalization to drive inflammatory macrophage phenotypic switch. The protein-protein docking and molecular dynamics simulation methods are the only computational approaches to predict these interactions and the stability of complexes formed among NF1, GLUT1, and Akt2. However, confirmation of these interactions requires site-directed mutagenesis. Relevant to these interactions, Akt2 inhibition for persons with NF1 is a complicated approach in that Akt1 appears to take on some of the putative Akt2 signaling in the absence of neurofibromin. These data imply that targeting the Akt2-GLUT1 pathway for persons with NF1 needs to be macrophage specific and incorporate combination therapies targeting PI3K and/or both Akt isoforms.

Finally, we provide compelling animal and human evidence that macrophage-mediated inflammation is critical to NF1-related angiogenesis. First, neurofibromin is sensitive to hypoxia. Following exposure to hyperoxia from P7 to P12, animals returned to normoxia experience a relative hypoxia that drives vascularization of the retina, with peak neovascularization occurring at P17. We demonstrate that retinal neurofibromin expression is suppressed during the hypoxia phase of OIR, and this corresponds to an increase in retina macrophage density and specifically increases inflammatory macrophage content within the retinal neovascular tuft. Although persons with NF1 have characteristic neovascularization of the retina and choroid that are now part of the diagnostic criteria for NF1,^24,34–36^ the implications of these findings extend beyond the retina since perivascular cuffing is a common feature of NF1 tumors. Further, we demonstrate that the relationship between over-expression of GLUT1 and loss of neurofibromin is conserved at the tissue level since GLUT1 expression is increased nearly 2-fold in *Nf1*^+/−^ retinas when compared with WT retinas despite macrophages representing a small fraction of the cell pool within the retina. Similar results are observed in human NF1 tumors with high concordance of GLUT1 and Akt2 within tumor macrophages.

In conclusion, we demonstrate that loss of neurofibromin facilitates GLUT1 translocation to the membrane of macrophages to facilitate a phenotypic switch between classically reparative and inflammatory macrophages. This relationship is enhanced via Akt2, which is more easily bound to GLUT1 in the absence of neurofibromin and activates its glucose internalization properties. These mechanistic findings provide a rational explanation for the high numbers of inflammatory macrophages observed within NF1 tumors and support a role for inflammatory macrophages in promoting neovascularization in the retina and tumors of persons with NF1.

## RESOURCE AVAILABILITY

### Lead contact

Correspondence and requests for further information should be directed to and will be fulfilled by the lead contact, Brian K. Stansfield (bstansfield@augusta.edu)

### Materials availability

This study did not generate new materials or unique reagents.

### Data and code availability

- Uncropped original western blot images, microscopy images, and raw data reported in this paper will be shared by the lead contact upon written request.
- This paper does not report original code.
- Any additional information required to reanalyze the data reported in this paper is available from the lead contact upon request.

## STAR★METHODS

### EXPERIMENTAL MODEL AND STUDY PARTICIPANT DETAILS

#### Sex as a biological variable

Both male and female mice were used for all *in vitro* and *in vivo* studies. Tissue samples from both male and female persons with NF1 were analyzed. Sex was not considered as a biologic variable for any studies.

#### Cells

Murine bone marrow-derived macrophages (BMDM), and human THP-1-derived macrophages were cultured in high glucose Dulbecco’s modified Eagle’s Medium (DMEM) medium containing 10% FBS, 100 U/mL of penicillin G and 100 mg/mL streptomycin. THP1-derived macrophages were kindly gifted from Dr. Gabor Csanyi (Augusta University, Augusta, GA). Media was supplemented with either Macrophage colony stimulating factor (M-CSF; 20 ng/mL, Invitrogen; PMC2044) or 20% L929 cell-derived conditioned media to differentiate bone marrow-derived monocytes into macrophages. Cells were cultured in the absence of M-CSF or L929 cell-derived conditioned media for at least 24 h prior to experiments. Human THP-1 monocytes were differentiated using PMA (200nM, Sigma; P1585). Both BMDM and THP-1 macrophages were polarized into so-called inflammatory macrophages (M^LPSIFNγ^) using IFNγ (20 ng/ml, PeproTech; 315–05) and LPS (100 ng/ml, CST; 14011) and reparative macrophages (M^IL4^) using IL-4 (20 ng/ml, PeproTech; 214–14) for 16 h in serum-free high glucose DMEM media.

#### Primary bone marrow-derived macrophages

Murine bone marrow monocytes were isolated and differentiated as described by Trouplin et al. with modifications.^38^ Briefly, the femur and tibia were dissected, cleaned, and flushed with sterile PBS using a 10mL syringe and a 25-gauge needle. Bone marrow was spun down and resuspended in differentiation media (high-glucose DMEM, 10% FBS, 20% L929 cells-derived conditioned media or M-CSF (20 ng/mL) and 1% penicillin/streptomycin). After thoroughly being pipetted to obtain a single cell suspension, the bone marrow was plated on 3 × 10cm petri dishes (uncoated). This was determined to be day 1. On day 3, the media was removed from the plates and replaced with fresh differentiation media. On day 7, differentiation media was replaced with polarization DMEM media containing IFNγ (20 ng/ml) and LPS (100 ng/ml) for inflammatory (M^LPSIFNγ^) and IL-4 (20 ng/mL), for reparative (M^IL4^) macrophages and were incubated for 16 h. On day 8, the macrophages were ready to use for experiments.

#### Animals

Mice were housed in the animal facility of Augusta University under a 12-h light/dark cycle with *ad libitum* access to food and water. *Nf1*-heterozygous (*Nf1*^+/−^), *Nf1*^*flox/flox*^;LysMCre^+^ (*Nf1*^ΔMΦ^) and mice were obtained from Tyler Jacks, Ph.D., (Massachusetts Institute of Technology, Cambridge, MA). *Nf1*^+/−^ mice were backcrossed 13 generations into the C57BL/6J strain. *Nf1*^*flox/flox*^;LysMCre^−^ (*Nf1*^fl/fl^) mice were obtained from Luis Parada (University of Texas Southwestern Medical Center, Dallas, TX, USA). *Nf1*^f/f^ and LysMCre^+^ mice were crossed till second generation to produce *Nf1*^f/f^ and *Nf1*^ΔMΦ^ mice which were used as control and myeloid cell-restricted knock out mice in experiments, respectively. Mice were genotyped by PCR as described.^39^ WT and *Nf1*^+/−^ mice (C57BL/6J) were crossed to generate experimental progeny. Littermate male mice, between 8 and 12 weeks of age, were used for experiments. All animal studies were performed in compliance with the Association for Research in Vision and Ophthalmology (ARVO) Statement for the Use of Animals in Ophthalmic and Vision Research and were approved by the Institutional Animal Care and Use Committee of Augusta University (Augusta, GA).

#### Human patient samples

The NF1 patient tissues (*n* = 5 tumors from 4 patients) were diagnosed and collected at Emory University. All surgeries were performed following the relevant guidelines and regulations at Emory University. The approval number of the present study by ethics committee is STUDY00002544. Written consent was obtained from all patients. After each surgery, the tissues were harvested, post-fixed in 4% paraformaldehyde overnight, and cut into blocks for paraffin embedding. The tissues were cut at 8um thickness and used for further analysis.

### METHOD DETAILS

#### Knockdown of NF1 in THP-1 macrophages

*NF1* was knocked down in THP-1 macrophages with a modified protocol from Zhang et al.^24^ *NF1* small hairpin (sh) RNA and shCtrl lentiviral vectors were purchased from Sigma (TRCN0000238778 and SHC202; St. Louis, MO, USA) and lentiviral supernatant was generated from Lenti-X 293T packaging cells. Cells were plated onto tissue culture plates and transfected the following day using Lipofectamine 3000 (Invitrogen; L3000001) with a combination of the vector plasmids (TRCN0000238778 or SH202), the 2^nd^ generation packaging plasmid psPAX2 (Addgene; 12260) and the envelope expressing plasmid pMD2.G (Addgene; 12259) in Opti-MEM for 3 h in the ratio of 2:6:4, respectively according to a protocol described by Batori et al.^40^ The transfection media was replaced with DMEM supplemented with 10% FBS and 2mM caffeine, with penicillin and streptomycin. Viral stocks were harvested 72 h after transfection, clarified by centrifugation, filtered through a 0.45-μm filter and concentrated using a sucrose gradient and stored at −80°C. THP1 macrophages were seeded onto tissue culture plates and transduced with lentiviral stock (20uL) in the presence of 8 μg/mL polybrene (Sigma) for 16 h. The next day 1mL of fresh media was added on top for 24hours. Fresh medium with a second dose of virus (20uL) without polybrene was added for another 48 h after which fresh medium with 1 μg/mL puromycin (ThermoFisher Scientific) was replaced for 3 days. Selected cells were cultured and *NF1* knockdown was confirmed by Western blot.

#### Seahorse XF glycolysis stress test

Cellular bioenergetics were measured using an XF96 analyzer (Agilent Seahorse XF Technology, Santa Clara, CA). Macrophages (transduced THP-1 with shCtrl and sh*NF1*, and BMDM from *Nf1*^fl/fl^ and *Nf1*^ΔMΦ^ mice) were seeded equally at 50,000 cells per well of 96-well plate and 24 h later polarizing cytokines (refer section Primary bone marrow-derived macrophages, *n* = 4 mice/genotype, 3–4 technical replicates/mice or per condition) were added and incubated for 16 h. Extracellular Acidification Rate (ECAR) as a measure of glycolysis in polarized transduced THP-1 cells and BMDM was measured using Seahorse XF glycolysis stress test kit (Agilent Technologies; 103020–100) as per manufacturer’s protocol. After 1 h of equilibration of transduced THP-1 cells and BMDM in DMEM, glycolysis (ECAR) was measured with injections of glucose (10 mM), oligomycin (1.5 μM) and 2-DG (50 mM). The report for glycolysis stress test was generated automatically using Seahorse XF Stress Test Report Generators. At the end of the experiment, the protein content in each well of 96-well plate was quantified to ensure accurate normalization of the ECAR data.

#### Glucose uptake assay

Glucose uptake was measured using a fluorescent 2-NBDG Glucose Uptake Assay Kit (BioVision; K682–50) as per manufacturer’s instruction with slight modifications. Briefly, polarized BMDM (M^CSF^, M^LPSIFNγ^ and M^IL4^) derived from long bones of *Nf1*^f/f^ and *Nf1*^ΔMΦ^ mice were seeded at a density of 500,000/well in 24-well plate in high glucose DMEM with 10% FBS (*n* = 3 mice/genotype). After 24 h, cells were serum starved in 0.2% serum rich DMEM for 30 min. Starved cells were then incubated with and without BAY-876 inhibitor (Cayman Chemical Company; 19961, 50nM) in 0.2% serum-rich DMEM for 30 min more. After incubation of BAY-876, cells were incubated with a FITC-labeled glucose analog (2-deoxy-2-[(7-nitro-2,1,3-benzoxadiazol-4-yl) amino]-D-glucose) for 30 min at 37°C and fluorescence was measured at a wavelength of 488 nm with flow cytometry.

#### RNA isolation, cDNA synthesis and quantitative Real-time PCR (qRT-PCR)

Total RNA from polarized BMDM (M^CSF^, M^LPSIFNγ^, M^IL4^) from *Nf1*^f/f^ and *Nf1*^ΔMΦ^ mice (*n* = 4 mice/genotype, 2 technical replicates/mouse) were extracted using RNeasy Mini kit (Qiagen; 74104). The resultant sample of total RNA (500ng-1μg) was utilized as a template for complementary DNA (cDNA) synthesis using either iScript cDNA synthesis kit (Bio-Rad; 1708891) or high-capacity M-MLV reverse transcriptase (Invitrogen; 28025013). qRT-PCR was performed using cDNA (diluted 1 in 10 with nuclease free water), on a StepOne Plus System (Applied Biosystems) or ABI 7500 Real-Time PCR System (Applied Biosystems, Foster City, CA) (Applied Biosystems, Foster City, CA, US), using iTaq Universal SYBR Green Supermix (Bio-Rad, 1725121) with the gene-specific primers.^41,42^ The primers used in this study were either obtained from TaqMan gene expression assay (Applied Biosystems) or Integrated DNA Technologies (Coralville, IA, US) (Table S3). Quantification of relative gene expression was calculated with the efficiency-corrected 2^−ΔΔCT^ method using murine *Gapdh* or *Rplp*0 or *Hprt* as the internal controls for normalization following Livak et al. method^43^ and data were presented as fold change relative to control groups. Details of primer sequences used for genes are provided in Table S3.

#### Protein extraction and western blot

Whole protein lysates were prepared from single cell suspension of BMDM from *Nf1*^f/f^ and *Nf1*^ΔMΦ^ mice (*n* = 3 mice/genotype) or cell lines or tissue with 1X Pierce RIPA buffer (ThermoFisher Scientific, 89900) supplemented with 1% proteinase/phosphatase inhibitor cocktail following established protocol.^44,45^ Protein concentration was determined by Pierce bicinchoninic acid (BCA) protein assay (ThermoFisher Scientific, 23227) according to the manufacturer’s instructions. Equal amount of protein (20mg) was loaded on 7.5%, or 10%, or 12% TGX Stain-free FastCast SDS-PAGE gel (Bio Rad, 1610181, 1610183 and 1610185) and transferred to PVDF membrane (Bio-Rad, 1620177). Primary antibodies used in this study were as follows: Rabbit GLUT-1 (abcam; ab115730, 1:100,000), Rabbit Na/K ATPase (abcam; ab76020, 1:10,000), Neurofibromin (Bethyl; A300–140A, 1:1000), Mouse GAPDH (Santa Cruz; sc-365062), Rabbit phospho-PI3K p85 (Tyr458)/p55 (Tyr199) (CST; 4228, 1:1000), Rabbit PI3K (CST; 4292, 1:1000), Rabbit phospho-Akt-1 (Ser473) (D7F10) XP (CST; 9018, 1:1000), Rabbit Akt-1 (CST; 2938, 1:1000), Rabbit phospho-Akt-2 (Ser474) (D3H2) (CST; 8599, 1:1000), Rabbit Akt-2 (D6G4) (CST; 3063, 1:1000), Rat F4/80 (Bio-Rad, MCA497R). Membranes were incubated with HRP-conjugated secondary antibodies for 1 h at room temperature. Membranes were developed using Pierce ECL western blotting substrate or SuperSignal West Femto Maximum Sensitivity Substrate (ThermoFisher Scientific, USA; 34095). Images were taken with the ChemiDoc MP system (Bio-Rad), and band densities were quantified in triplicates using Image Lab software (Bio-Rad). Data were normalized either by total protein or GAPDH as loading control.

#### Subcellular protein fractionation of BMDM

To determine the facilitative GLUT1 translocation and P-Akt-2 expression in membrane and cytoplasm, subcellular fractionation of polarized BMDM from *Nf1*^f/f^ and *Nf1*^ΔMΦ^ mice was performed (*n* = 3 mice/genotype) using Subcellular protein fractionation kit for cultured cells (ThermoFisher Scientific; 78840) according to manufacturer’s protocol with slight modifications. Briefly, BMDM at a density of 10 million for each condition (M^CSF^, M^LPSIFNγ^ and M^IL4^) were seeded in 10 cm plates, differentiated for 7 days using differentiation media, and polarized using specific cytokines into so called M^LPSIFNγ^ and M^IL4^ macrophages (refer section *Isolation of mouse macrophages, differentiation and polarization*). Cytoplasmic fractions were isolated first by incubating cells in cytoplasmic extraction buffer for 15 min at 4°C, followed by collection of membrane fractions using membrane extraction buffer for 15 min at 4°C. Both fractions were used later for immunoblotting.

#### Co-immunoprecipitation

To determine the complex formation between NF1 and GLUT1 in BMDM from *Nf1*^f/f^ mice, co-immunoprecipitation was performed using immunoprecipitation kit (ThermoFisher Scientific; 10007D) according to manufacturer’s protocol with slight modifications. Briefly, the BMDM from *Nf1*^f/f^ mice were isolated, differentiated and polarized to M^LPSIFNγ^ and M^IL4^ macrophages (refer section Primary bone marrow-derived macrophages). Cells were subsequently washed once with PBS, followed by lysis in 2X RIPA buffer with 1% proteinase/phosphatase inhibitor cocktail to make lysates. Firstly, 50μL of Dynabeads were incubated with 8ug of GLUT1 antibody (CST, 12939) for 2 h at 4°C on rotation. The Dynabeads-antibody complex made was clarified using DynaMag-2 Magnet (ThermoFisher Scientific; 12321D), followed by washing in antibody binding and washing buffer by gentle pipetting. This Dynabead-antibody complex was incubated with lysates (800ug of protein) overnight at 4°C on rotation to allow the antigen to bind the Dynabeads-antibody complex. Next day, Antigen-Dynabeads-antibody complex was washed in washing buffer and separated. The Antigen-Dynabeads-antibody complex was resuspended in 2X sample buffer and heated for 10 min at 70°C. The complex was loaded onto the gel for immunoblotting. Primary antibody used in this study is Neurofibromin (Bethyl; A300–140A, 1:1000). Secondary HRP-conjugated antibody or TidyBlot HRP-conjugated (Bio-Rad; STAR209PA, 1:400) were used to develop the bands.

#### Protein-protein docking

The X-ray crystal structures of glucose transporter 1 (GLUT1: PDB ID code 4PYP), Akt2 (PDB ID code 8Q61), and neurofibromin 1 (NF1:PDB ID code 8K4L) obtained from the Protein DataBank (PDB). Prior to docking, all heteroatoms present in the structures were removed. The modeling process was performed using the HDOCK server (http://hdock.phys.hust.edu.cn/) which employs computational algorithms to predict the three-dimensional structures of protein complexes based on known interactions and structural data. The knowledge-based iterative scoring function, ITScorePP or ITScorePR, determines the docking scores in Hdock. Since the docking score has not been calibrated to the experimental data, it should not be interpreted as the genuine binding affinity between two molecules; rather, a higher negative docking score indicates a more plausible binding model.

#### Atomistic simulations

Molecular dynamics simulations were conducted using Gromacs package 2018.1. Initially the protein-protein complex of GLUT1-Akt2 was setup with amber99sb-ILDN forcefield in a cubic box whose sides were kept 1nm apart from complex. The model was solvated using TIP3P water molecules and subjected to energy minimization through 5,000 steps of the steepest-descent method. To neutralize the systems, counterions were added. Subsequently, a second round of energy minimization was conducted with an additional 5,000 steps of the steepest-descent method. Two rounds of equilibration were performed: firstly, a 0.1 ns equilibration at constant number, volume, and temperature using a time step of 0.002 ns, employing the V-rescale algorithm for temperature coupling at 310 Kelvin with a coupling time constant of 0.5 ps, separately applied to proteins/complexes, and solvent; secondly, a 1 ns equilibration at constant number, pressure, and volume with a time step of 0.002 ns, utilizing Nose–Hoover temperature coupling and Parrinello–Rahman pressure coupling. The system’s pressure was semi-isotropically coupled using the Berendsen algorithm at 1 bar, with a compressibility of 4.5 × 10^−5^ and a coupling time constant of 5.0 ps. The production run was conducted for 250 ns without any restraints, employing a time step of 0.002 ns. A neighbor-list generation and Coulomb and Lennard-Jones interactions cutoff of 1.2 nm were selected, with Particle–Mesh–Ewald summation employed for electrostatic interactions. End-state free energy calculations with GROMACS were conducted by using a tool gmx_MMPBSA.^37^

#### Oxygen-induced retinopathy

To induce retinal pathological angiogenesis in *Nf1*^f/f^ and *Nf1*^ΔMΦ^ mice, the OIR model was used, as previously described.^46^ Nursing dams with postnatal day 7 (P7) pups were housed in a sealed chamber and exposed continuously to 70% oxygen. At P12, the animals were returned to room air (21%). Pups with body weight less than 4 g at P17 were excluded from analysis. Retinas from P17 pups were collected for experiments. Data from six pups per litter were used for all experiments.

#### Retina dissection and immunofluorescence staining

For quantification of retinal neovascularization in retina whole mount at P17 OIR stage, eyes from *Nf1*^f/f^ and *Nf1*^ΔMΦ^ pups exposed to oxygen were enucleated and fixed in 4% PFA/PBS overnight at 4°C (*n* = 10 retinas/genotype). The retinas were dissected out, washed in PBS for 10 min in a shaker. Retinas were then permeabilized and blocked in blocking solution (10% donkey serum and 1% Triton X-100 in PBS) for 1 h at room temperature. After washing in PBS, retinas were stained with Alexa Fluor 594-conjugated Isolectin GS-IB_4_ (Invitrogen; I21413, 1:100 dilution in 3% donkey serum with 0.3% Triton X-100) overnight at 4°C. Next day after washing three times in PBS, the retinas were mounted on glass slides using mounting media without DAPI (Vector Laboratories; H-1000). Images were acquired at 20X magnification with Leica Stellaris confocal microscope. Quantification of avascular area and neovascular tufts was performed in Adobe Photoshop, as previously described by Connor et al.^47^

For whole-mount immunofluorescence staining for macrophage/microglia at P17 OIR stage, eyes from *Nf1*^f/f^ and *Nf1*^ΔMΦ^ pups (*n* = 6 retinas/genotype) were processed, fixed and permeabilized similarly as for Isolectin-B4 staining. Fixed retinas were then stained with Alexa Flour-conjugated primary antibodies prepared in blocking solution (3% goat serum and 0.3% Triton X-100 in PBS). Primary antibodies were used as follows: Alexa Fluor 594-F4/80 (Biolegend; 123140, 1:100), Rat anti-mouse APC-CD86 (BD Biosciences; 558703, 1:50), Alexa Fluor 647-CD206 (Biolegend; 141712, 1:100) and Alexa Fluor 488-conjugated Isolectin GS-IB_4_ (Invitrogen; I21411, 1:100). Images were acquired at higher magnification (40X and 60X) with Leica Stellaris confocal microscope. Pearson’s correlation coefficient for co-localization of F4/80 with CD86 or CD206 was calculated using Just Another Colocalization plugin (JA-CoP) in ImageJ software.

#### Flow cytometry

Cells were stained according to the protocol described previously by Zaidi et al.^48^ Briefly, retinas from *Nf1*^f/f^ and *Nf1*^ΔMΦ^ P17 OIR pups (n = 5–6 mice/genotype; total of 10–12 retinas/genotype) were collected and single cell suspension from both retinas together was made by gentle mechanical dissociation in ice-cold staining buffer (BD Biosciences; 554656). Cells were centrifuged, pellet were collected and assessed for viability using Zombie NIR fixable viability kit (BioLegend; 423105) for 30 min at 4°C. Cells were then incubated in FcR (CD16/CD32) Blocking reagent (BD Biosciences; 553141) for 20 min at 4°C. For surface marker staining, cells were incubated with fluorescently conjugated primary antibodies, PerCP-CD11b (BioLegend; 101230) and FITC-F4/80 (BioLegend; 123108) for 30 min at 4°C in dark, followed by washing with ice-cold staining buffer and centrifuged at 300g for 5 min. For intracellular staining, cells were permeabilized and fixed with BD Cytofix/Cytoperm buffer (BD Biosciences; 555028) for 20 min at 4°C in dark, followed by washing with BD Perm/Wash buffer (BD Biosciences; 555028). Cells were washed, centrifuged at 300g for 5 min and were stained with primary antibodies, PE-Cy7-iNOS (Invitrogen; 25–5920-82) and PE-Arg-1 (Invitrogen; 12–3697-82) for 30 min at 4°C in dark. Finally, cells suspension was washed, centrifuged and filtered in 5mL tube with cell strainer cap (40um) and analyzed on Acea NovoCyte Penteon flow cytometer (Agilent). Novocyte software was used to analyze the data. Unstained and Fluorescence minus one (FMO) controls were used for gating. Compensation was done using UltraComp eBeads plus compensation beads (Invitrogen; 01–3333-42).

#### Immunofluorescent staining of human tissue neurofibromas

Paraffin-waxed human neurofibroma sections were stained as previously described with slight modifications.^48^ Briefly, to retrieve antigen epitopes, heat-mediated antigen retrieval (target retrieval solution, Dako) was performed. Sections were then rinsed in PBS-T (1X PBS with 0.25% Triton X-100), blocked in goat/donkey serum for 1 h. Slides were incubated with primary antibodies; mouse anti-mouse CD68 (Santa Cruz; sc-17832, 1:100), and rabbit anti-mouse P-Akt2 (S474) (D3H2) (CST; 8599, 1:100) overnight at 4°C on shaker. The following day, slides were washed and incubated with Alexa Fluor-conjugated secondary antibodies; goat anti-mouse Alexa Fluor 568 (Invitrogen A-21144, 1:300) and goat anti-rabbit Alexa Fluor 647 (Invitrogen; A-21244, 1:300) for 1 h at room temperature, respectively. In between primary antibody incubations, slides were blocked again with blocking buffer for 1 h to inhibit non-specific binding. Slides were then incubated with primary antibody; rabbit anti-mouse GLUT-1 (CST; 12939, 1:100) overnight and the next day, fluorescent secondary antibody corresponding to GLUT-1 (Alexa Fluor 488 donkey anti-rabbit; Invitrogen; A21206, 1:300) was added and incubated for 1 h at room temperature. Nuclei were visualized by mounting medium with DAPI (Vector Laboratories; H-1200). Images were acquired at higher magnification (40X) with Leica Stellaris confocal microscope. A representative image of at least 4 patients were shown.

### QUANTIFICATION AND STATISTICAL ANALYSIS

P-values were calculated by One-way ANOVA followed by Tukey’s or Sidak’s or Dunnette’s post hoc test for multiple comparisons and two-tailed Student’s t-test was used to compare pairs of data groups using GraphPad Prism. In all figures, data are expressed as mean ± SD values. Figures representing *in vitro* technical replicates are presented as mean ± SEM values; (*p < 0.05, **p < 0.01, ***p < 0.001, ****p < 0.0001, ns, not significant).

## Supplementary Material

1

SUPPLEMENTAL INFORMATION

Supplemental information can be found online at https://doi.org/10.1016/j.celrep.2025.115625.

## Figures and Tables

**KEY RESOURCES TABLE:** 

REAGENT or RESOURCE	SOURCE	IDENTIFIER

Antibodies		

PerCP anti-mouse/human CD11b	BioLegend	Cat# 101230; RRID: AB_2129374
FITC anti-mouse F4/80	BioLegend	Cat# 123108; RRID: AB_893502
PE-Cy7 anti-mouse iNOS	Invitrogen	Cat# 25-5920-82; RRID: AB_2573499
PE anti-mouse/human Arg-1	Invitrogen	Cat# 12-3697-82; RRID: AB_2734839
Glucose Transporter GLUT1 [EPR3915]	Abcam	Cat# ab115730; RRID: AB_10903230
Sodium Potassium ATPase [EP1845Y]	Abcam	Cat# ab76020; RRID: AB_1310695
Rabbit anti-mouse NF1	Bethyl	Cat# A300-140A; RRID: AB_2149790
Mouse anti-mouse GAPDH	Santa Cruz Biotechnology	Cat# sc-365062; RRID: AB_10847862
Rabbit anti-mouse phospho-PI3 Kinase p85 (Tyr458)/p55 (Tyr199)	Cell Signaling Technology	Cat# 4228; RRID: AB_659940
Rabbit anti-mouse/human/rat PI3 Kinase	Cell Signaling Technology	Cat# 4292; RRID: AB_329869
Rabbit phospho-Akt-1 (Ser473) (D7F10) XP	Cell Signaling Technology	Cat# 9018; RRID: AB_2629283
Rabbit anti-mouse Akt-1	Cell Signaling Technology	Cat# 2938; RRID: AB_915788
Rabbit phospho-Akt-2 (Ser474) (D3H2)	Cell Signaling Technology	Cat# 8599; RRID: AB_2630347
Rabbit Akt-2 (D6G4)	Cell Signaling Technology	Cat# 3063; RRID: AB_2225186
Rat anti-mouse F4/80	Bio-Rad	Cat# MCA497R; RRID: AB_323279
Rabbit anti-mouse Glucose Transporter GLUT1	Cell Signaling Technology	Cat# 12939; RRID: AB_2687899
Alexa Fluor 594 anti-mouse F4/80	BioLegend	Cat# 123140; RRID: AB_2563241
APC Rat anti-mouse CD86	BD Biosciences	Cat# 558703; RRID: AB_2075114
Alexa Fluor 647 anti-mouse CD206	BioLegend	Cat# 141712; RRID: AB_10900420
Goat anti-mouse Alexa Fluor 568	Invitrogen	Cat# A-21144; RRID: AB_2535780
Goat anti-rabbit Alexa Fluor 647	Invitrogen	Cat# A-21244; RRID: AB_2535812
Donkey anti-rabbit Alexa Fluor 488	Invitrogen	Cat# A21206; RRID: AB_2535792

Bacterial and virus strains		

N/A	N/A	N/A

Biological samples		

Human: NF1 patient’s tumor tissues	Emory University	Approval number # STUDY00002544
Mouse: Retina samples from *Nf1*^+/−^, *Nf1^f/f^* and *Nf1*^ΔMΦ^ mice	This paper	N/A
Mouse: bone marrow-derived macrophages from *Nf1^f/f^* and *Nf1*^ΔMΦ^ mice	This paper	N/A

Chemicals, peptides, and recombinant proteins		

Macrophage colony stimulating factor (M-CSF)	Invitrogen	Cat# PMC2044
Phorbol 12-myristate 13-acetate (PMA)	Sigma-Aldrich	Cat# P1585
Lipopolysaccharide (LPS)	Selleckchem	Cat# S7850
Murine IFN-gamma (IFNγ)	PeproTech	Cat# 315-05
Murine IL-4	PeproTech	Cat# 214-14
Akt-2 inhibitor (CCT128930; Akt2i)	Selleckchem	Cat# S2635
GLUT1 inhibitor; BAY-876 inhibitor	Cayman Chemical Company	Cat# 19961
FcR (CD16/CD32) Blocking reagent	BD Biosciences	Cat# 553141
BD Cytofix/Cytoperm buffer	BD Biosciences	Cat# 555028
BD Perm/Wash buffer	BD Biosciences	Cat# 555028
1X Pierce RIPA buffer	ThermoFisher Scientific	Cat# 89900
7.5% TGX Stain-free FastCast SDS-PAGE gel kit	Bio Rad	Cat# 1610181
10% TGX Stain-free FastCast SDS-PAGE gel kit	Bio Rad	Cat# 1610183
12% TGX Stain-free FastCast SDS-PAGE gel kit	Bio Rad	Cat# 1610185
SuperSignal West Femto Maximum Sensitivity Substrate	ThermoFisher Scientific	Cat# 34095
TidyBlot HRP-conjugated	Bio-Rad	Cat# STAR209PA
Alexa Fluor 594-conjugated Isolectin GS-IB_4_	Invitrogen	Cat# I21413
Mounting media without DAPI	Vector Laboratories	Cat# H-1000
Mounting medium with DAPI	Vector Laboratories	Cat# H-1200

Critical commercial assays		

Seahorse XF glycolysis stress test kit	Agilent Technologies	Cat# 103020-100
Fluorescent 2-NBDG Glucose Uptake Assay Kit	BioVision	Cat# K682-50
Viability using Zombie NIR fixable viability kit	BioLegend	Cat# 423105
Pierce bicinchoninic acid (BCA) protein assay	ThermoFisher Scientific	Cat# 23227
RNeasy Mini kit	Qiagen	Cat# 74104
iScript^™^ cDNA synthesis kit	Bio-Rad	Cat# 1708891
High-capacity M-MLV reverse transcriptase	Invitrogen	Cat# 28025013
iTaq Universal SYBR Green Supermix	Bio-Rad	Cat# 1725121
Subcellular protein fractionation kit	ThermoFisher Scientific	Cat# 78840
Immunoprecipitation kit	ThermoFisher Scientific	Cat# 10007D

Deposited data		

X-ray crystal structures of glucose transporter 1 (GLUT1)	This paper	PDB: 4PYP
X-ray crystal structures of Akt2	This paper	PDB: 8Q61
X-ray crystal structures of neurofibromin 1 (NF1)	This paper	PDB: 8K4L

Experimental models: Cell lines		

Mouse: L929 Fibroblast cell (NCTC clone 929)	ATCC	Cat# CCL-1; RRID: CVCL_0462
Mouse: Bone marrow-derived macrophages	This paper	N/A
Human: THP-1 cells	Gabor Csanyi’s Laboratory	N/A
Human: Lenti-X^™^ 293T packaging cells	David Fulton’s Laboratory	N/A

Experimental models: Organisms/strains		

Mouse: *Nf1*-heterozygous (*Nf1*^+/−^)	Dr. Tyler Jacks	Massachusetts Institute of Technology, Cambridge, MA
Mouse: *Nf1^flox/flox^*; LysMCre^−^ (*Nf1*^fl/fl^)	Dr. Luis Parada	University of Texas Southwestern Medical Center, Dallas, TX, USA
Mouse: *Nf1^flox/flox^*;LysMCre^+^ (*Nf1*^ΔMΦ^)	Dr. Tyler Jacks	Massachusetts Institute of Technology, Cambridge, MA
Mouse: C57BL/6J	The Jackson Laboratory	000664, Jackson Laboratories, Bar Harbor, ME
Mouse: LysMCre+	The Jackson Laboratory	00472, Jackson Laboratories, Bar Harbor, ME

Oligonucleotides		

Primers for *Mcp1*, *Nos2, Il10, Il1b, Nfkb, Tnfa, Rplp0, Arg1*, *Il6, Glut1*, *Hk1, Pfkfb3, Ldha, Hprt*	This paper	see Table S3

Recombinant DNA		

MISSION^®^ TRC2 pLKO.5-puro non-target shRNA Control Plasmid DNA	Sigma-Aldrich	Control Plasmid # SHC202
pLKO.1-puro -CMV-Turbo GFP-*NF1* shRNA Plasmid DNA	Sigma-Aldrich	Custom Plasmid # TRCN0000238778
psPAX2	Addgene	Packaging Plasmid RRID: Addgene_12260
pMD2.G	Addgene	Expression Plasmid RRID: Addgene_12259

Software and algorithms		

Novocyte flow cytometer software	Agilent	https://www.agilent.com/en/product/research-flow-cytometry/flow-cytometry-software/novocyte-novoexpress-software-1320805
Image Lab Software for Mac Version 6.1 SOFT-LIT-170-9690-ILSMAC-V-6-1	Bio-Rad	https://www.bio-rad.com/en-us/product/image-lab-software?ID=KRE6P5E8Z
HDOCK server	This paper	(http://hdock.phys.hust.edu.cn/)
GROMACS	gmx_MMPBSA^37^	
Seahorse XF Stress Test Report Generators	Agilent	https://www.agilent.com/en/product/cell-analysis/real-time-cell-metabolic-analysis/xf-software
Adobe Photoshop	This paper	https://www.augusta.edu/it/software.php
Adobe Illustrator	This paper	https://www.augusta.edu/it/software.php
ImageJ software	This paper	https://imagej.net/ij/
GraphPad Prism 10	GraphPad Prism Software	https://www.graphpad.com/features

Other		

UltraComp eBeads plus compensation beads	Invitrogen	Cat# 01-3333-42

## Highlights

- Reparative macrophages lacking neurofibromin exhibit increased glycolysis
- Loss of neurofibromin facilitates GLUT-1 translocation to the membrane via Akt2
- Loss of neurofibromin promotes inflammatory macrophage phenotypic switch
- Neurofibromin-deficient macrophages accumulate near vessels and promote angiogenesis
