# Supplementary material for: Loss of neurofibromin induces inflammatory macrophage phenotypic switch and retinal neovascularization via GLUT1 activation
Source: Cell Rep. Author manuscript; Available in PMC 2025 Jun 23. (PMC12184535; doi:10.1016/j.celrep.2025.115625)
Supplement: 1 [file NIHMS2085636-supplement-1.pdf]

Cell Reports, Volume *44*

Supplemental information

Loss of neurofibromin induces inflammatory macrophage phenotypic switch and retinal neovascularization via GLUT1 activation

Yusra Zaidi, Rebekah Tritz, Nida Zaidi, Faisal Nabi, Syed Adeel H. Zaidi, Abdelhakim Morsy, Valerie Harris, Rilee Racine, Farlyn Z. Hudson, Zsuzsanna Bordan, Simone Kennard, Robert Batori, Yuqing Huo, Gabor Csanyi, Eric J. Belin de Chantemèle, Kecheng Lei, Nicholas M. Boulis, David J. Fulton, Rizwan Hasan Khan, Ruth B. Caldwell, and Brian K. Stansfield

SUPPLEMENTAL INFORMATION

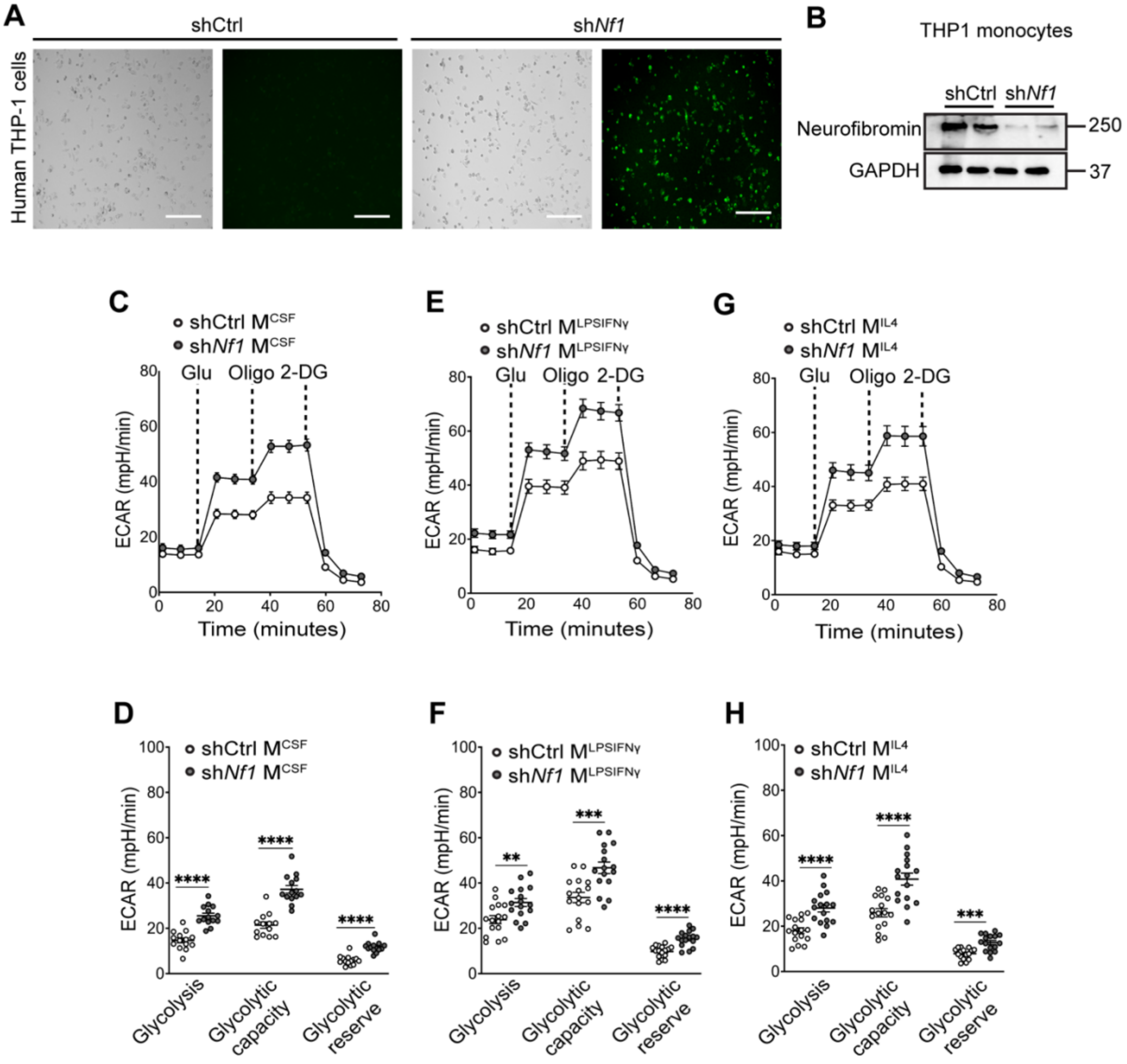

Figure S1. *Nf1* knockdown increases glycolysis in human THP1 macrophages.
(**A**) Representative fluorescent microscope images showing human THP1 monocytes transduced (GFP green signal) with control shRNA (shCtrl) and *Nf1* shRNA (sh*Nf1*) for 16 hours, followed by puromycin selection for 3 days. After puromycin selection, THP1 monocytes were differentiated by PMA (200nM) and polarized using 20ng/mL LPS and IFN-γ (for inflammatory macrophages; $M^{LPSIFN\gamma}$) or 20ng/mL IL-4 (for reparative macrophages; M^{IL4}). Scale bars: 100μm. (**B**) Representative immunoblot showing confirmation of *Nf1* knockdown in human THP1 monocytes after transduction with shCtrl and sh*Nf1*. (**C-H**) Representative measurements of Extracellular acidification rate (ECAR) performed on THP1 macrophages transduced with shCtrl and sh*Nf1* RNA, differentiated with PMA (200nM) and polarized with 20ng/mL LPS and IFN-Y or 20ng/mL IL-4 for inflammatory ($M^{LPSIFN\gamma}$) or reparative (M^{IL4}) macrophages for 16 hours, respectively. Subsequent addition of glucose, the ATP synthase inhibitor oligomycin, and the hexokinase inhibitor 2-deoxy-glucose (2-DG) were carried out where indicated (**C, E** and **G**), n = 14 technical replicates/condition. Data are expressed as mean ± SEM. P values were calculated using One-way ANOVA followed by Dunnett's T3 multiple comparison test (*P < 0.05, ** P < 0.01, *** P < 0.001, **** P < 0.0001, ns, not significant).

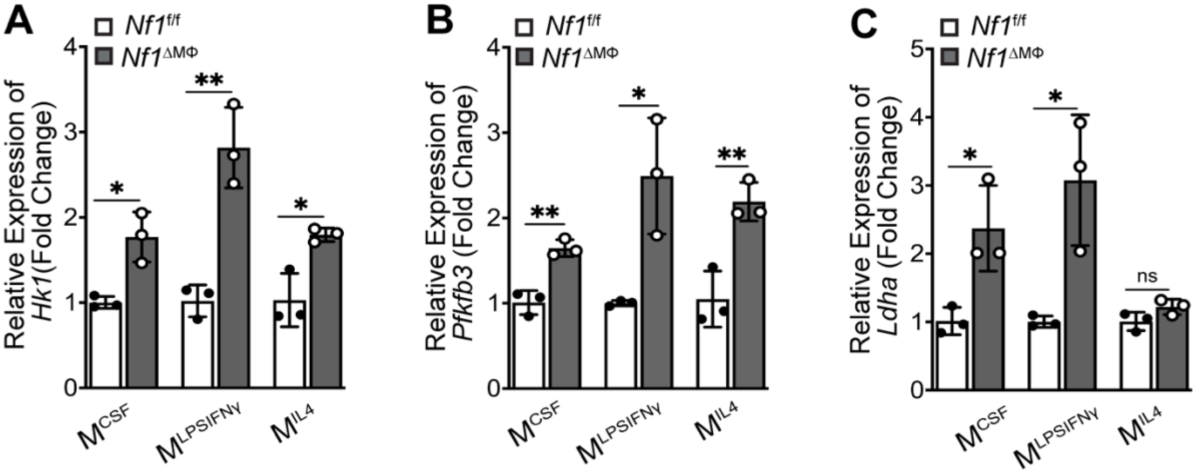

Figure S2. Loss of neurofibromin increases mRNA expression of potent glycolysis stimulators in inflammatory ($M^{LPSIFN\gamma}$) and reparative (M^{IL4}) macrophages. Quantitative RT-PCR data showing relative mRNA expression of (**A**) Hexokinase1 (*Hk1*), (**B**) 6-phosphofructo-2-kinase/fructose-2,6-bisphosphatase (*Pfkfb3*) and (**C**) Lactate dehydrogenase-a (*Ldha*) in control (M^{CSF}), inflammatory ($M^{LPSIFN\gamma}$) and reparative (M^{IL4}) macrophages-derived from long bones of *Nf1*$^{f/f}$ and *Nf1*$^{\Delta M\Phi}$ mice. *Hprt* is considered as endogenous control in this study (n=3 mice per genotype, each data point represents the mean of two technical replicates of each mouse). Data are expressed as mean ± SD. P values calculated using 2-tailed Student's t-test (*P < 0.05, ** P < 0.01, *** P < 0.001, **** P < 0.0001, ns, not significant).

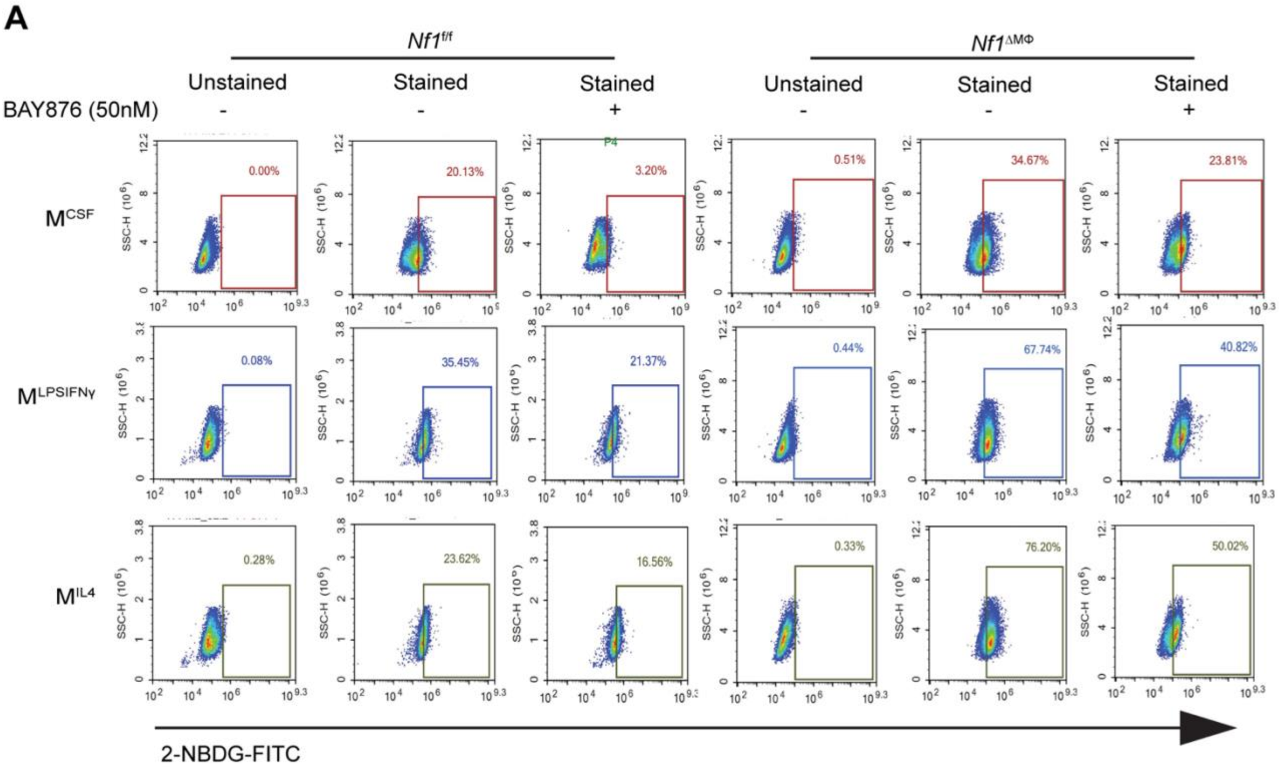

Figure S3. Gating strategy showing percent glucose uptake by bone marrow-derived macrophages (Related to Figure 1G-J). (**A**) Representative flow cytometric plots of percent glucose uptake by viable BMDMs derived from bones of $Nf1^{f/f}$ and $Nf1^{\Delta M\varphi}$ mice in absence or presence of GLUT1 inhibitor, BAY876. BMDMs were labeled with 2-NBDG at a concentration of 100μM for 30 minutes in absence or presence of BAY876 (50nM), to measure glucose uptake by flow cytometry, n = 3 mice per genotype.

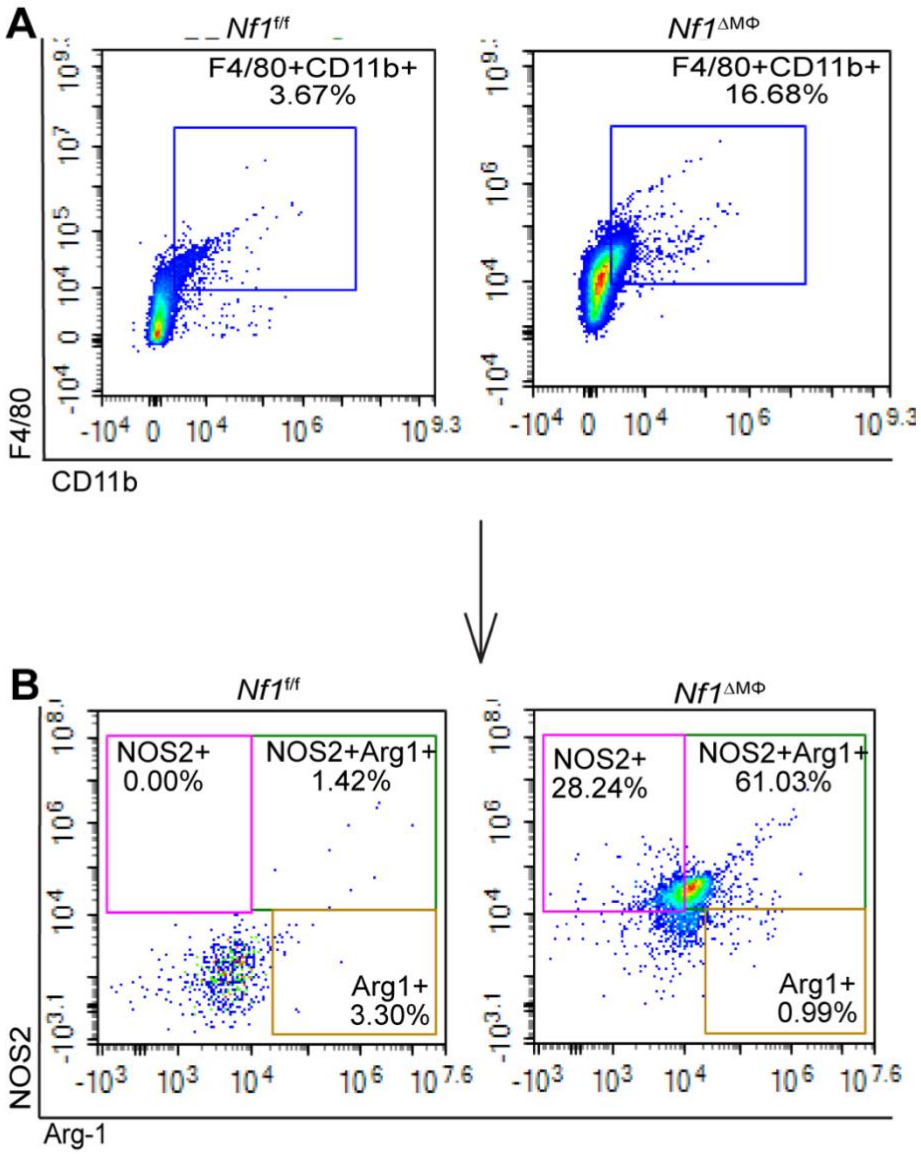

Figure S4. Gating strategy showing Macrophage/microglia in P17 OIR retinas from $Nf1^{f/f}$ and $Nf1^{\Delta M\varphi}$ pups exhibit mixed phenotype (inflammatory + reparative phenotype) (**Related to Figure 6A-C**). (**A**) Representative dot plots show gating of CD11b+/F4/80+ macrophage/microglia in P17 OIR retinas. (**B**) The selected CD11b+/F4/80+ cells were analyzed for intracellular markers NOS2 (for inflammatory) and Arginase1 (reparative) by flowcytometry. n = 5-6 pups/genotype, total 10-12 retinas in each genotype**.**

Table S1: Molecular docking of GLUT1 with NF1 and AKT2 by H-DOCK (**Related to Figure 4**).

Neurofibromin		GLUT1		GLUT1	AKT2	
	GLU2123A	VAL173		THR60	ARG371	
	GLU2123A	LEU176		THR63	ARG371	
	GLU2123A	TYR308		LEU64	ARG371	
	GLU2123A	ILE311		LEU67	ARG243	
	THR2124A	VAL173		VAL74	ARG347	
	THR2124A	ILE179		ILE78	ARG245	
	GLN2126A	LEU169		ILE78	GLY346	
	VAL2127A	ILE170		ILE78	ARG347	
	VAL2127A	VAL173		GLY79	ARG245	
	VAL2127A	PHE174		PHE81	TYR351	
	LEU2130A	VAL166		PHE81	GLN353	
	LEU2130A	ILE170		SER82	ARG245	
	GLU2134A	LEU199		LEU85	PHE238	
	LEU2137A	MET13		LEU85	SER242	
	LEU2137A	GLY17		PHE86	SER242	
	PRO2138A	GLY10		PHE86	ARG243	
	PRO2138A	MET13		PHE86	ARG245	
	PRO2138A	LEU14		**ARG89**	PHE239	
	LYS2139A	LEU14		**PHE90**	PHE239	
	LYS2139A	LEU21		**MET98**	ARG243	
	LYS2139A	LEU199		MET99	SER242	
	LYS2139A	VAL203		MET99	ARG243	
	PHE2140A	LEU14		MET99	ARG245	
	PHE2140A	LEU199		**LEU101**	ARG243	Protein Kinase Domain
	LEU2142A	LEU14		**LEU101**	GLU244	
	LEU2143A	LEU199		LEU102	ARG243	
	LEU2143A	ILE202		LEU102	GLU244	
	THR2179A	ILE202		LEU102	ARG245	
	SER2180A	ILE202		GLU120	ARG371	
	GLU2182A	LEU198		GLU120	THR372	
	GLU2182A	ILE202		ILE123	THR372	
	THR2183A	LEU198		LEU124	VAL246	
	THR2183A	LEU199		LEU124	THR372	
	THR2183A	ILE202		PHE127	ARG245	
	GLU2186A	PHE194		PHE127	CYS345	
	GLU2186A	ILE195		PHE127	GLY346	
	GLU2186A	LEU198		PHE127	ARG347	
	GLU2190A	PHE174		ILE128	GLU244	
	GLU2190A	ILE195		ILE128	ARG245	
	ARG2197A	SER178		ILE128	VAL246	
	ARG2197A	ILE179		VAL131	ARG245	
	ARG2197A	MET180		TYR132	ARG245	
	ARG2197A	LEU185		LEU135	ARG245	
	LYS2279A	LEU198		ILE259	GLN353	
	PRO2282A	PHE112		LEU260	GLN353	
	LEU2283A	PHE112		LEU260	HIS355	
	GLU2318A	**LEU101**		PHE263	GLN353	
	HIS2322A	**LEU101**		PHE263	ASP354	

	HIS2322A	PHE104		ARG264	ASP354	
	HIS2322A	VAL105		ARG264	HIS355	
	THR2323A	VAL108		ARG264	GLU356	
	SER2326A	VAL108		ALA405	GLN353	
	SER2326A	LEU109		VAL406	GLN353	
	SER2326A	MET121		PHE409	TYR351	
	SER2381A	PRO205		PHE409	ASN352	
	PRO2382A	ARG93		PHE409	GLN353	
	PRO2382A	ASN94		**ARG89**	TYR438	AGC-Kinase C Terminal Domain
	PRO2382A	GLU209		**ARG89**	PHE439	
	ALA2383A	ARG93		**ARG89**	ASP440	
	ALA2383A	ASN94		**ARG89**	ASP441	
	ALA2383A	LEU97		**PHE90**	ARG437	
	ALA2383A	PRO205		**PHE90**	ASP441	
	ALA2386A	ASN94				
	ALA2386A	**MET98**				
	VAL2389A	**MET98**				
	ARG2390A	LEU97				
	ARG2390A	**MET98**				
	ARG2390A	MET99				
	ARG2390A	**LEU101**				
	VAL2424A	**ARG89**				
	VAL2424A	**PHE90**				
	MET739B	LYS255				
	GLU740B	ARG253				
	SER743B	ARG253				
	VAL744B	ARG253				
	LYS757B	ARG249				
	ARG758B	ARG249				
	ARG758B	ARG253				
	ALA761B	ARG249				
	ALA761B	ARG253				
	LEU762B	ARG253				
	ARG765B	GLN250				
	ARG765B	ARG253				
Ras GAP Domain (CHAIN B)	ARG1375B	ILE339				
	ARG1375B	ALA342				
	ARG1375B	PHE378				
	PHE1376B	PHE378				
	PRO1377B	PHE320				
	GLN1378B	VAL316				
	GLN1378B	PHE320				
	GLN1378B	VAL370				
	GLN1378B	PHE373				
	GLN1378B	GLY374				
	GLN1378B	ALA377A				
	ASN1379B	VAL370A				
	HIS1431B	TYR366A				
	VAL1432B	TRP363A				
	LEU1433B	TRP363A				
	PHE1434B	TRP363A				
	THR1435B	LEU361A				
	THR1435B	TRP363A				

	ARG1441B	GLN360A		
	ARG1441B	LEU361A		
	ARG1441B	PRO362A		

Table S2. Summary of human NF1 patients selected for neurofibromas tissues sectioning for immunofluorescent staining of macrophages, GLUT1 and P-Akt2 (**Related to Figure 7**).

Study ID	Age (Years)	Sex	NF1	Location
NF1-12	27	F	Y	L Brachial Plexus
NF1-19	32	M	Y	R Posterior Thigh
NF1-21	38	F	Y	R Mid-Calf
NF1-26	38	F	Y	C3-C4 Intradural
NF1-23	27	M	Y	R Retroperitoneal

Table S3: Primers for qRT-PCR used in the study (**Related to STAR Methods**).

Mice Gene ID	Sequence / Reference ID	Probes	Source
Mcp1	Forward: GGCTCAGCCAGATGCAGTTAA<br>Reverse: CCTACTCATTGGGATCATCTTGCT	SYBR Green	IDT
Nos2	Forward: GGCAGCCTGTGAGACCTTTG<br>Reverse: TGCATTGGMGTGAAGCGTTT	SYBR Green	IDT
Il10	Forward: GCTCTTACTGACTGGCATGAG<br>Reverse: CGCAGCTCTAGGAGCATGTG	SYBR Green	IDT
Il1b	Forward: TGCCACCTTTTGACAGTGATG<br>Reverse: ATGTGCTGCTGCGAGATTTG	SYBR Green	IDT
Nfkb (Rela (p65)	Forward: TCCTGTTCGAGTCTCCATGCAG<br>Reverse: GGTCTCATAGGTCCTTTTGCGC	SYBR Green	IDT
Tnfa	Forward: GGTCCCCAAAGGGATGAGAA<br>Reverse: TGAGGGTCTGGGCCATAGAA	SYBR Green	IDT
Rplp0	Forward: CCTCCTTCTTCCAGGCTTTG<br>Reverse: CCACCTTGTCTCCAGTCTTTATC	SYBR Green	IDT
Arginase-1	Mm00475988-m1	FAM	TaqMan Assay
Il6	Mm00446190-m1	FAM	TaqMan Assay
Glut1	Forward: GCAGTTCGGCTATAACACTGG<br>Reverse: GCGGTGGTTCCATGTTTGATT)	SYBR Green	IDT
Hk1	Forward: AACGGCCTCCGTCAAGATG<br>Reverse: GCCGAGATCCAGTGCAATG	SYBR Green	IDT
Pfkfb3	Forward: GATCTGGGTGCCCGTCGATCACCG<br>Reverse: CAGTTGAGGTAGCGAGTCAGCTTC	SYBR Green	IDT
Ldha	Forward: ACATTGTCAAGTACAGTCCACAC<br>Reverse: TTCCAATTACTCGGTTTTTGGGA	SYBR Green	IDT
Hprt	Forward: GAAAGACTTGCTCGAGATGTCATG<br>Reverse: CACACAGAGGGCCACAATGT	SYBR Green	IDT
